# Enhanced Proton Spillover at Pt‐Cluster/NiO Interface Reduces the Acidic–Alkaline Hydrogen Evolution Activity Gap

**DOI:** 10.1002/smsc.202500627

**Published:** 2026-01-28

**Authors:** Ashwani Kumar, Jinsun Lee, Min Gyu Kim, Harun Tüysüz

**Affiliations:** ^1^ Department of Heterogeneous Catalysis Max‐Planck‐Institut für Kohlenforschung Kaiser‐Wilhelm‐Platz 1 Mülheim an der Ruhr 45470 Germany; ^2^ Beamline Research Division Pohang Accelerator Laboratory (PAL) Pohang 790‐784 South Korea; ^3^ Catalysis and Energy Materials IMDEA Materials Institute Calle Eric Kandel 2 Getafe Madrid 28906 Spain

**Keywords:** electrocatalysis, hydrogen evolution, oxygen‐defect, proton spillover, water dissociation

## Abstract

The sluggish hydrogen evolution reaction (HER) kinetics in alkaline media, primarily attributed to the additional water dissociation step, has led to a significant activity gap between acidic and alkaline conditions. Metal‐supported electrocatalysts leveraging hydrogen spillover have garnered significant attention due to sufficiently utilized reaction sites; however, designing active catalysts remains a formidable challenge, primarily due to the limited understanding of the specific regulatory mechanisms governing proton spillover. Herein, a facile strategy is reported for the fabrication of Pt nanoclusters (Pt_NC_) on oxygen‐defect‐rich NiO nanowires (Pt_NC_‐D‐NiO). The electrocatalyst demonstrates excellent intrinsic and mass‐normalized HER activity and remarkable long‐term stability, outperforming Pt_NC_ on pristine NiO nanowires and commercial Pt/C. Notably, its alkaline HER activity is fairly close to its acidic counterpart, significantly narrowing the activity gap compared to commercial Pt/C. Advanced ex situ*/*operando physicochemical characterizations, including in situ electrochemical impedance spectroscopy, reveal that oxygen defects substantially lower the water dissociation energy barrier. This facilitates rapid H* spillover and enhances local H* coverage on Pt_NC_, thus accelerating subsequent H* recombination to boost alkaline HER. This work not only offers a cost‐effective catalyst design strategy but also provides fundamental insights into the role of hydrogen spillover in optimizing electrocatalytic performance.

## Graphical abstract

The integration of Pt nanoclusters onto oxygen‐deficient NiO significantly accelerates both the water dissociation and proton transfer steps via the hydrogen spillover mechanism, thereby narrowing the activity gap between alkaline and acidic hydrogen evolution reactions.

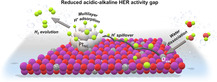

## 1 Introduction

The electrocatalytic hydrogen evolution reaction (HER), powered by renewable electricity, provides a sustainable pathway for green hydrogen (H_2_) production and a promising alternative to fossil fuels.^[^ ^1^, ^2^ ^]^ The design and development of an efficient and cost‐effective HER electrocatalyst in both acidic and alkaline electrolyte have been the focus of extensive research for decades.^[^ ^3^ ^]^ An ideal HER catalyst should facilitate optimized hydrogen intermediate (H*) adsorption on the catalyst surface, adhering to Sabatier's principle (neither too weak nor too strong).^[^ ^4^ ^]^ In alkaline media, the cathodic HER is further hindered by the sluggish water dissociation process (Volmer step) required to generate H*, followed by the hydrogen combination process (Heyrovsky or Tafel step).^[^ ^5^ ^]^ At present, platinum‐based materials are regarded as the benchmark electrocatalyst for HER due to their near‐optimal H* adsorption Gibbs free energy (Δ*G*_H_ ≈ 0); however, their scarcity significantly limits widespread and sustainable applications.^[^ ^2^ ^]^ To enhance Pt utilization and reduce catalyst costs, downsizing Pt‐species to nanoclusters could expose enough active sites while preserving the intrinsic metallic Pt–Pt interactions.^[^ ^6^ ^]^ Therefore, the development of Pt‐based HER electrocatalysts with nanocluster dimensions is crucial for accelerating reaction kinetics and achieving superior catalytic performance.

Although Pt, with its near‐optimal H* adsorption energy, is the most active catalyst for acidic HER, its catalytic kinetics in alkaline media are at least two orders of magnitude slower.^[^ ^7^ ^]^ This limitation primarily arises from its insufficient water dissociation capability, which hinders proton supply for H_2_ generation. In contrast, transition metal‐based oxides are considered ideal catalysts for the oxygen evolution reaction due to their strong water adsorption ability; however, their HER activity remains poor due to significantly positive Δ*G*_H_, leading to decreased H* adsorption and sluggish water dissociation kinetics.^[^ ^8^, ^9^ ^]^ A well‐designed electrocatalyst for pH‐universal HER, particularly in alkaline media, should feature distinct active sites for water dissociation and hydrogen adsorption steps to take place, respectively, ensuring efficient reaction kinetics (Figure S1, Supporting Information). Guided by this principle, hydrogen spillover‐based binary component catalysts have emerged as a promising class of HER electrocatalysts, predominantly comprising metal–support systems.^[^ ^10^, ^11^, ^12^ ^]^ Beyond serving as a stabilizing matrix for active metal sites, the support plays a crucial role in modulating metal–support interactions, thereby enhancing overall reaction kinetics. An ideal hydrogen spillover‐based pH‐universal HER catalyst should integrate sites that facilitate water adsorption/dissociation, a component with near‐optimal hydrogen adsorption/desorption energy (Δ*G*_H_ ≈ 0), and an efficient H* spillover pathway between them. Recently, oxygen vacancies in metal oxides have been identified as key factors in promoting water adsorption/dissociation, thereby improving the activity of hydrogen spillover‐based catalysts.^[^ ^13^, ^14^, ^15^ ^]^ For instance, oxygen vacancy‐rich Pt–TiO_2_ has been shown to facilitate reverse charge transfer, which enhances hydrogen spillover and promotes H* reduction on electron‐rich Pt sites.^[^ ^16^ ^]^ Cho et al. demonstrated the synergistic optimization of oxygen vacancies and elemental substitution in Ru/P–TiO_2_, boosting water dissociation and H* desorption.^[^ ^17^ ^]^ A similar effect was also observed in NiMo alloys,^[^ ^18^ ^]^ Rh/MoS_2_,^[^ ^19^ ^]^ and Pt/WO_3_^[^ ^20^ ^]^ for electrocatalytic HER. Despite recent advances, most studies failed to fully elucidate the detailed mechanism of hydrogen spillover, particularly in alkaline media, and have overlooked the critical H* transfer step in the spillover process. Therefore, it is essential to develop a straightforward strategy to integrate Pt‐based nanoclusters on modified metal oxide supports, facilitating favorable hydrogen spillover and unlocking their full potential for catalyzing HER in both acidic and alkaline conditions.

Herein, we report an efficient hydrogen‐spillover strategy to enhance the H* transfer step by integrating Pt‐nanocluster onto oxygen‐defect‐rich ordered mesoporous NiO (Pt_NC_‐D‐NiO), thereby improving HER kinetics in both acidic and alkaline media. The strongly anchored Pt_NC_ serve as the H* adsorption/combination site, while the adjacent oxygen‐defect‐rich NiO facilitates the sluggish water dissociation step, thus increasing local proton coverage via interfacial hydrogen spillover in alkaline conditions. The as‐prepared Pt_NC_‐D‐NiO exhibited excellent pH‐universal HER intrinsic activity, mass activity, and long‐term stability, particularly in alkaline media, outperforming Pt_NC_ on pristine NiO and commercial Pt/C. Moreover, the Pt_NC_‐D‐NiO significantly narrows the HER kinetic gap between acidic and alkaline electrolytes compared with those of commercial Pt/C. Mechanistic investigations using various experimental techniques and operando characterizations confirm that both the Pt_NC_ and the defect‐rich NiO play vital and synergistic roles in activating alkaline HER, thereby reducing the activity gap relative to acidic HER.

## 2 Results and Discussion

### 2.1 Synthesis and Structural Characterization

We developed a facile two‐step strategy to fabricate binary‐component electrocatalysts by integration Pt_NC_ onto ordered mesoporous pristine/defective NiO support (see Experimental Section for details). The ordered mesoporous (nanocasted) NiO support was synthesized via a nanocasting method using SBA‐15 as a hard template. Powder X‐ray diffraction (XRD) confirmed formation of pristine NiO (P‐NiO) with diffraction peaks corresponding to a pure NiO face‐centered‐cubic (FCC) phase (JCPDS 47‐1049) (Figure S2, Supporting Information).^[^ ^21^, ^22^ ^]^ Transmission electron microscopy (TEM) images show a highly ordered nanowire morphology in P‐NiO (Figure S3a, Supporting Information). To obtain defective NiO (D‐NiO) enriched with oxygen vacancies (O_vac_), the calcination atmosphere was switched from air to argon while preserving the ordered nanowire structure and the NiO FCC phase (Figure S2 and S3b, Supporting Information).^[^ ^14^, ^23^ ^]^ The inert atmosphere during calcination limits oxygen availability, thereby promoting the formation of oxygen defects within the NiO lattice, as reported in previous studies.^[^ ^14^, ^23^, ^24^ ^]^ Raman spectroscopy further supports the presence of oxygen vacancies in defective NiO (D‐NiO), as evidenced by redshifts in the one‐phonon transverse optic (1P‐TO ≈ 461 cm^−1^) and one‐phonon longitudinal optic (1P‐LO ≈ 495 cm^−1^) modes compared to P‐NiO, consistent with prior literature (Figure S4, Supporting Information).^[^ ^14^, ^25^, ^26^ ^]^

**Figure 1:**
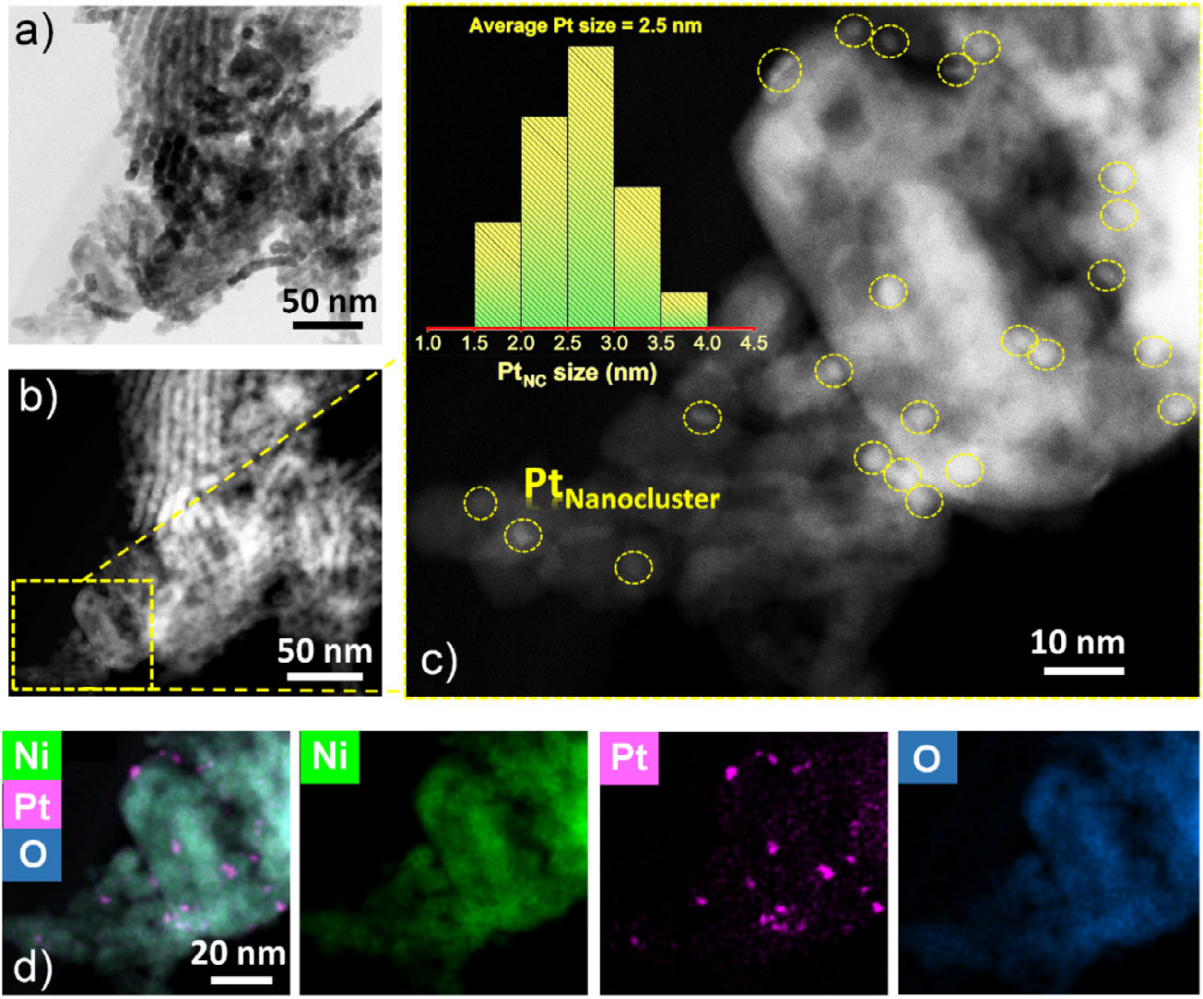
Electron microscopy of the sample. a) TEM and b) HAADF‐STEM image of Pt_NC_‐P‐NiO. c) Magnified HAADF‐STEM image of Pt_NC_‐P‐NiO in panel (b). Inset: the size distribution of Pt nanoclusters. d) Corresponding elemental mapping images of Pt_NC_‐P‐NiO.

Subsequently, the P‐NiO/D‐NiO supports were soaked in an ethanolic Pt‐ion solution for 1 h, followed by annealing at 350 °C for 2 h to form Pt_NC_ on P‐NiO (Pt_NC_‐P‐NiO) and D‐NiO (Pt_NC_‐D‐NiO). The morphology and crystal phase of the resulting materials remained similar to those of their respective substrates, indicating the existence of Pt as amorphous nanoclusters (**Figure** **1**a, Figure S2 and S5a, Supporting Information). Furthermore, the Pt_NC_‐D‐NiO still exhibited a higher content of oxygen‐defect sites compared to Pt_NC_‐P‐NiO, as revealed by the respective Raman analysis (Figure S4, Supporting Information). Energy‐dispersive X‐ray spectroscopy (EDS) and inductively coupled plasma optical emission spectrometry (ICP‐OES) analyses further confirmed Pt loading of ≈2.4 ± 0.1 and ≈2.4 ± 0.3 wt% on the surfaces of P‐NiO and D‐NiO, respectively (Figure S6 and Table S1, Supporting Information).

High‐angle annular dark‐field scanning transmission electron microscopy (HAADF‐STEM) images unambiguously confirm the presence of amorphous Pt sub‐nanoclusters on the surfaces of both P‐NiO and D‐NiO supports, with average cluster sizes of ≈2.5 and ≈2.2 nm, respectively, excluding the Pt‐size effects on their catalytic performance (Figure 1b,c and Figure S5b–d, Supporting Information). Moreover, the corresponding EDS elemental mapping analysis revealed the presence of Pt and its uniform dispersion across both the NiO support surfaces (Figure 1d, and Figure S5e, Supporting Information).

### 2.2 Spectroscopic Characterizations

The local coordination environment and electronic structure of Pt were further investigated using synchrotron‐based X‐ray absorption spectroscopy (XAS) and X‐ray photoelectron spectroscopy (XPS). The rising edge of the Ni K‐edge X‐ray absorption near‐edge structure (XANES) spectra of Pt_NC_‐D‐NiO displayed a slight negative shift in energy compared to Pt_NC_‐P‐NiO, indicating a reduction in the average oxidation state of Ni, which can be attributed to the introduction of oxygen‐defect sites under inert calcination conditions (Figure S7a, Supporting Information).^[^ ^27^ ^]^ The Fourier‐transformed *k* ^3^‐weighted extended X‐ray absorption fine structure analysis (FT‐EXAFS) spectra at the Ni K‐edge displayed dominant contributions from Ni—O and Ni—O—Ni scattering paths at 1.67 and 2.65 Å, respectively, corresponding to the first and second shells of NiO (Figure S7b, Supporting Information). Moreover, the Ni K‐edge FT‐EXAFS fitting analysis revealed that the first‐shell Ni—O coordination number (CN) for Pt_NC_‐D‐NiO was 4.6 at a fitted distance of 2.1 Å, lower than that of Pt_NC_‐P‐NiO (CN: 5.7; very close to the theoretical CN of 6 for standard NiO),^[^ ^22^ ^]^ aligning well with the observed Ni oxidation state variation and further corroborating the generation of oxygen‐deficient sites under inert calcination conditions, in agreement with Raman analysis results (Figure S8 and Table S2, Supporting Information).

**Figure 2:**
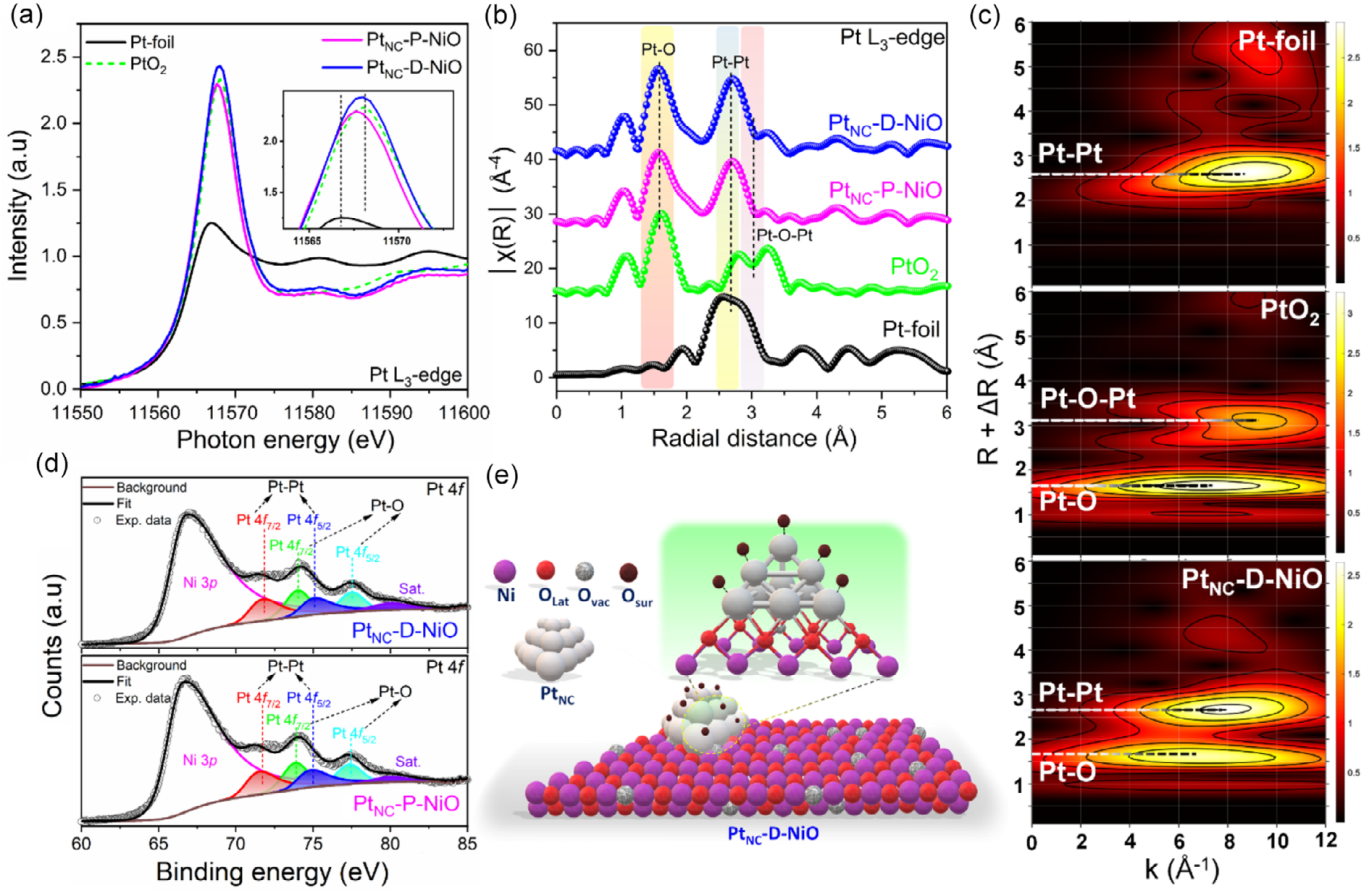
Electronic structure characterizations of the catalysts. a) Experimental Pt‐L_3_ edge XANES spectra of Pt_NC_‐D‐NiO and Pt_NC_‐P‐NiO with other reference samples (inset: the magnified white line feature). b) Experimental Pt‐L_3_ edge FT‐EXAFS spectra of Pt_NC_‐D‐NiO and Pt_NC_‐P‐NiO with other reference samples. c) Wavelet transform EXAFS (WT‐EXAFS) of Pt_NC_‐D‐NiO along with reference samples at Pt‐L_3_ edge. d) Fitted deconvoluted high‐resolution Pt 4*f* XPS spectra of Pt_NC_‐D‐NiO and Pt_NC_‐P‐NiO. e) Schematic illustration of Pt nanocluster anchored on defective NiO support.

The analysis of the Pt L_3_‐edge XANES spectra revealed that the white‐line maxima of Pt_NC_‐P‐NiO and Pt_NC_‐D‐NiO were positioned between those of Pt foil and PtO_2_, corresponding to Pt oxidation states of +2.5, and +2.8, respectively, indicating a strong electronic interaction between Pt_NC_ and NiO supports (**Figure** **2**a and Figure S9a, Supporting Information). In the Pt L_3_‐edge FT‐EXAFS spectra (Figure 2b), peaks observed at 1.6 and 2.7 Å were assigned to Pt—O and metallic Pt—Pt coordination, respectively, in both Pt containing samples. Notably, the absence of a Pt—O—Pt scattering path at ≈3.2 Å, which is characteristic of bulk PtO_2_, indicates the formation of ultrasmall metallic Pt_NC_. The presence of Pt—O coordination in both samples further implies that the sub‐nanocluster Pt_NCs_ are effectively integrated with the NiO support through bonding interactions with surface lattice oxygen, accompanied by surface oxidation of the Pt_NCs_. Consistently, the Pt L_3_‐edge wavelet transform‐EXAFS (WT‐EXAFS) analysis confirmed that the bonding environment of Pt_NC_‐P‐NiO and Pt_NC_‐D‐NiO were dominated by Pt—O and Pt—Pt coordination (Figure 2c, and Figure S9b, Supporting Information). The Pt L_3_‐edge XAS results of Pt_NC_‐P‐NiO and Pt_NC_‐D‐NiO were further corroborated by high‐resolution Pt 4*f* XPS analysis. As revealed in Figure 2d, the Pt_NC_ in both Pt containing samples exhibited contribution from both Pt—Pt (Pt^0^) and Pt—O (Pt^δ+^) interactions, aligning well with the Pt L_3_‐edge FT‐EXAFS analysis. Collectively, these structural and spectroscopic characterizations confirm the successful integration of partially oxidized amorphous Pt_NC_ onto oxygen‐defect rich NiO, as schematically illustration in Figure 2e, which is expected to significantly enhance HER catalytic kinetics under the demanding alkaline environment.

### 2.3 Electrocatalytic Performance toward HER

**Figure 3:**
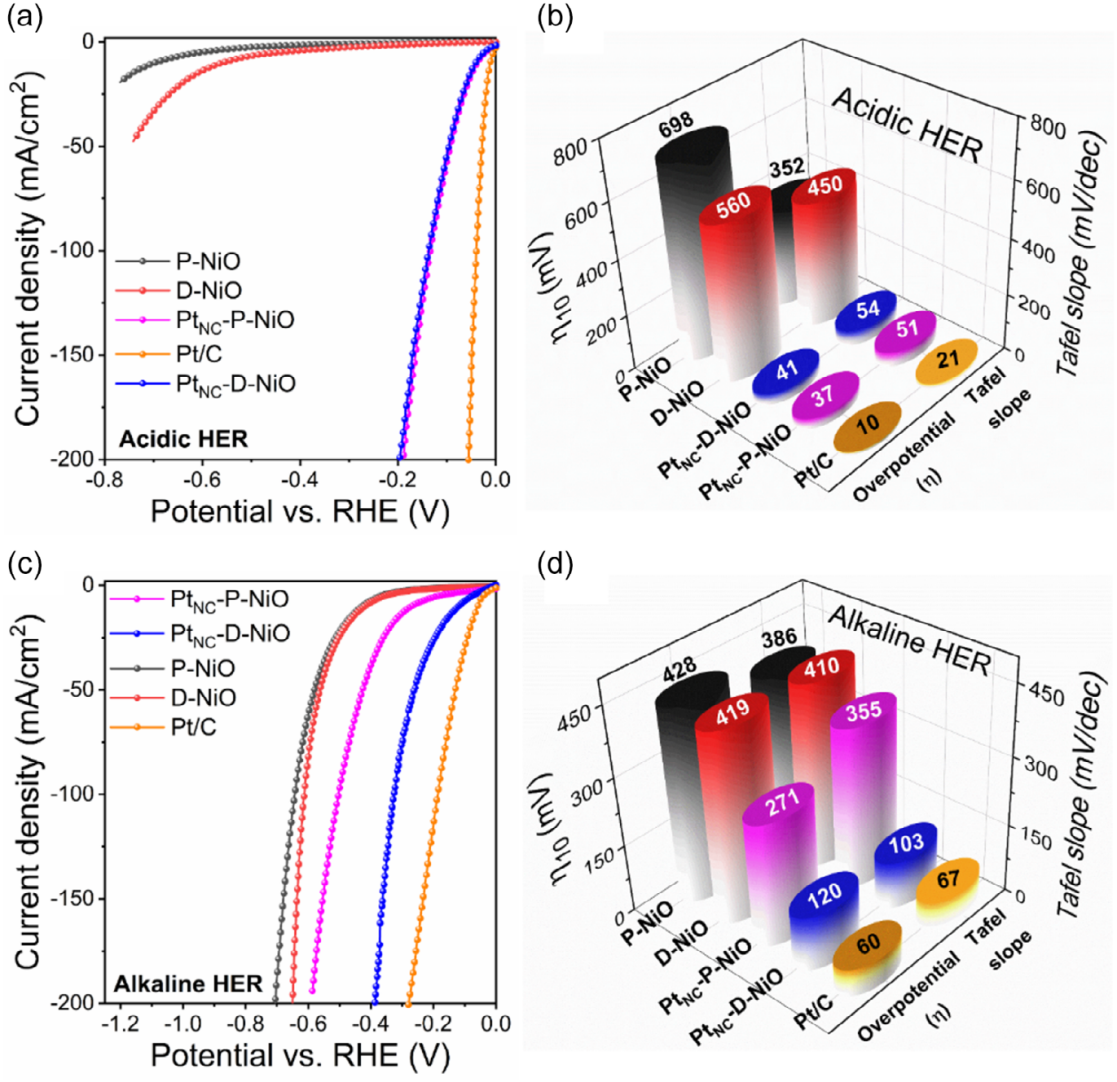
Electrochemical HER performance of catalysts. a) HER LSV polarization curves and b) the overpotentials required to reach −10 mA cm^−2^ and corresponding Tafel plots in 0.5 m H_2_SO_4_. c) HER LSV polarization curves and d) the overpotentials required to reach −10 mA cm^−2^ and corresponding Tafel plots in 1 m KOH.

The electrocatalytic HER activity of the synthesized heterostructures and commercial catalysts was systematically evaluated in both acidic (0.5 m H_2_SO_4_) and alkaline (1 m KOH) media using a standard three‐electrode setup to elucidate the structure–activity relationship.^[^ ^2^, ^28^ ^]^ The potential of the reference electrodes was calibrated in an H_2_ saturated electrolyte, and all potentials were referenced to the reversible hydrogen electrode (RHE) (Figure S10, Supporting Information).^[^ ^22^ ^]^ As depicted in the *iR*‐compensated linear sweep voltammetry (LSV) polarization curves in **Figure** **3**a, commercial Pt/C exhibited superior HER activity in acidic media, requiring a low overpotential (*η*) of only 10 mV to achieve −10 mA cm^−2^. In comparison, Pt_NC_‐P‐NiO and Pt_NC_‐D‐NiO demonstrated similar acidic HER activity, requiring *η*_10_ values of 37 and 41 mV, respectively, suggesting that the Pt_NCs_ with comparable cluster sizes and near‐optimal H* adsorption/desorption energy serve as the primary active sites for acidic HER (Figure 3b). This indicates that the P/D‐NiO support plays a minimal role in enhancing catalytic activity under acidic conditions. The corresponding Tafel plots derived from the LSV curves revealed that the Pt/C exhibited the smallest Tafel slope (21 mV dec^−1^), indicative of the Volmer–Tafel HER mechanism, where the electrochemical desorption step (Tafel step) serves as the rate‐determining step (RDS) (Figure 3b and Figure S11a,c, Supporting Information).^[^ ^29^ ^]^ Conversely, both Pt_NC_‐P‐NiO and Pt_NC_‐D‐NiO displayed comparable Tafel slope values (≈50–55 mV dec^−1^), suggesting a Volmer–Heyrovsky mechanism in which the electrochemical desorption (Heyrovsky step) is the RDS.^[^ ^30^ ^]^

In alkaline media, as expected, commercial Pt/C required a higher *η*_10_ (60 mV) at −10 mA cm^−2^ compared to acidic conditions (Figure 3c,d). Notably, Pt_NC_‐D‐NiO required an *η*_10_ of 120 mV, significantly lower than that of Pt_NC_‐P‐NiO (*η*_10_: 271 mV), highlighting the critical role of oxygen defect sites in D‐NiO toward facilitating water dissociation and enhancing proton availability for H_2_ generation. Moreover, Pt_NC_‐D‐NiO in alkaline HER exhibited a relatively low Tafel slope of 103 mV dec^−1^, comparable to Pt/C (67 mV dec^−1^), suggesting a Volmer–Heyrovsky HER mechanism where the electrochemical desorption (Heyrovsky step) is the RDS (Figure 3d, and Figure S11b,c, Supporting Information). In contrast, Pt_NC_‐P‐NiO displayed a significantly higher Tafel slope (355 mV dec^−1^), indicating that the HER follows the Volmer–Heyrovsky mechanism in which the sluggish Volmer step (water dissociation) is the RDS.^[^ ^30^, ^31^ ^]^ The enhanced HER activity of Pt_NC_‐D‐NiO compared to Pt_NC_‐P‐NiO, particularly in alkaline media, was further substantiated by its lower charge transfer resistance (*R*_CT_) and comparable electrochemically active surface area (ECSA), indicating accelerated charge transfer kinetics and an abundance of active sites, thereby facilitating superior overall HER performance (Figure S12 and S13, Supporting Information).

**Figure 4:**
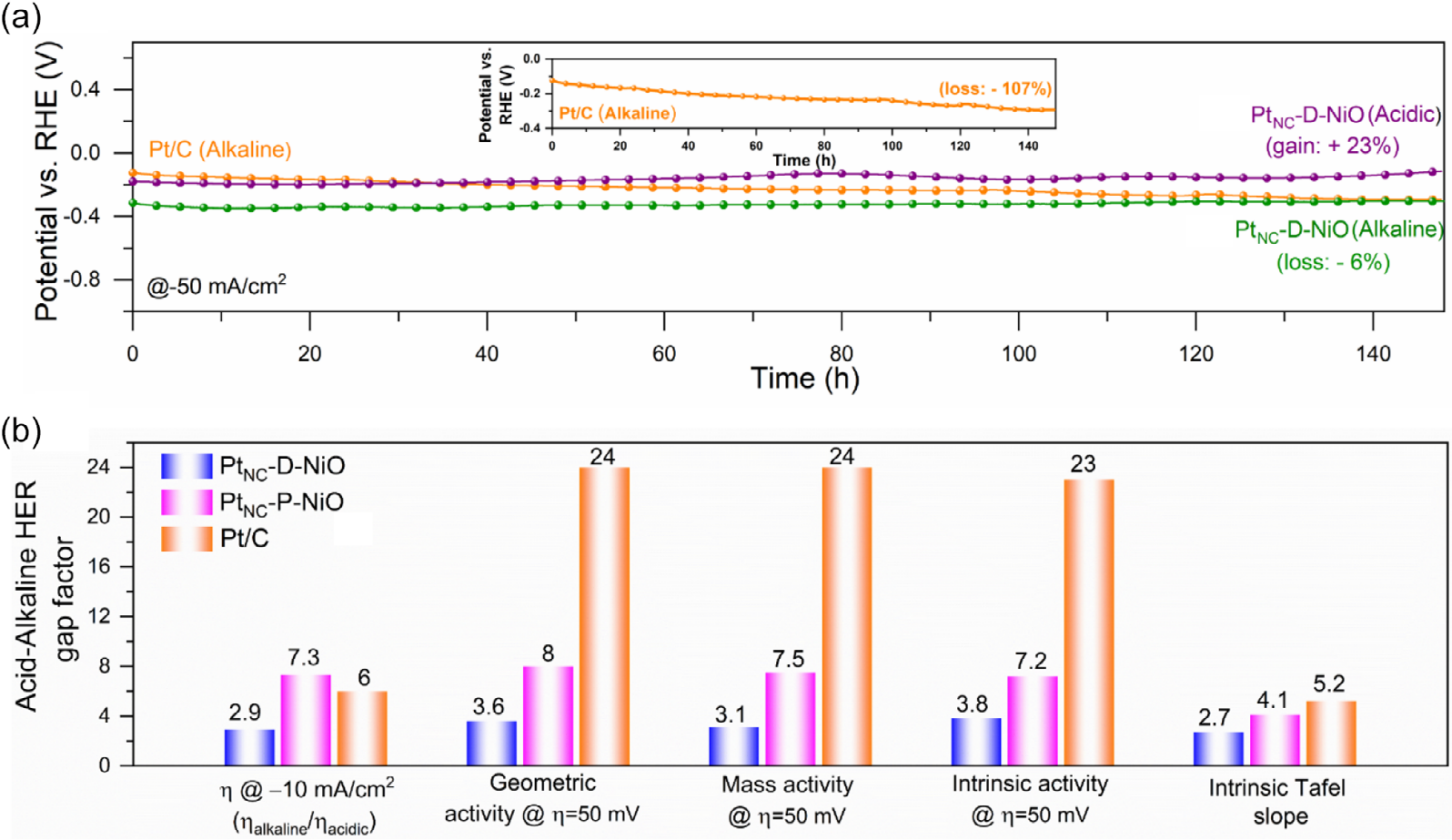
Durability test and acid–alkaline HER gap factor. a) Chronopotentiometric stability test for Pt_NC_‐D‐NiO in 0.5 m H_2_SO_4_ and 1 m KOH at a current density of −50 mA cm^−2^. The inset shows the stability test for Pt/C in 1 m KOH at −50 mA cm^−2^. b) Comparison of the acid–alkaline HER gap factor on overpotential, geometric activity, mass activity, intrinsic activity, and intrinsic Tafel slope. The HER gap factor can be determined by *η*_alkaline_/*η*_acid_, geometric activity_acid_/geometric activity_alkaline_, mass activity_acid_/mass activity_alkaline_, intrinsic activity_acid_/intrinsic activity_alkaline_, and intrinsic Tafel slope_alkaline_/intrinsic Tafel slope_acid_.

The long‐term durability of the catalysts in both acidic and alkaline media was evaluated using chronopotentiometry. The Pt_NC_‐D‐NiO displayed excellent long‐term stability in alkaline media, maintaining performance for 150 h at −50 mA cm^−2^ without any noticeable degradation, significantly surpassing the stability of commercial Pt/C (**Figure** **4**a). The nearly overlapping LSV curves recorded before and after the durability test further confirmed the retention of active sites and sustained high HER activity in alkaline media for Pt_NC_‐D‐NiO, unlike Pt/C (Figure S14, Supporting Information). Poststability characterization revealed that the crystal structure and composition of Pt_NC_‐D‐NiO remained intact, while the Pt L_3_‐edge XAS analysis indicated a reduction in the oxidation state of Pt_NC_, suggesting the elimination of surface oxidation under cathodic reduction conditions (Figure S15, Supporting Information). Moreover, Pt_NC_‐D‐NiO also exhibited a highly durable acidic HER performance (150 h at −50 mA cm^−2^); however, a notable enhancement in acidic HER activity was observed after the durability test, attributed to the oxidation and subsequent redeposition of Pt ions from the Pt counter electrode onto the working electrode under highly acidic conditions (Figure 4a and Figure S16, Supporting Information). To eliminate this influence, the Pt counter electrode was replaced with a graphite rod, resulting in minimal change in the acidic HER activity of Pt_NC_‐D‐NiO (Figure S17, Supporting Information). Furthermore, Pt_NC_‐D‐NiO demonstrated long‐term acidic HER stability, exhibiting negligible loss or gain in activity when the graphite rod was used as the counter electrode (Figure S18, Supporting Information). The pH‐universal HER performance (activity/stability) of Pt_NC_‐D‐NiO was comparable and better than many of the reported HER electrocatalysts (Table S3 and Figure S19, Supporting Information).

The geometric current density as a function of potential for Pt/C, Pt_NC_‐P‐NiO, and Pt_NC_‐D‐NiO in both acidic and alkaline HER is presented in Figure S20a (Supporting Information). Compared to Pt_NC_‐P‐NiO and Pt/C, Pt_NC_‐D‐NiO exhibits a notably different activity gap between acidic and alkaline HER in terms of the required *η*_10_ and geometric current density (Figure S20b,c, Supporting Information). Specifically, the geometric current densities of Pt_NC_‐P‐NiO and Pt/C in alkaline media are significantly lower than those in acidic media at an overpotential of 50 mV, whereas Pt_NC_‐D‐NiO demonstrates a much smaller geometric activity gap between the two media, highlighting its enhanced catalytic performance in alkaline HER. Furthermore, the HER mass activity was evaluated for Pt/C, Pt_NC_‐P‐NiO, and Pt_NC_‐D‐NiO by normalizing the acidic and alkaline HER catalytic current to their respective noble‐metal mass loading (Figure S21a,b, Supporting Information). Notably, Pt_NC_‐D‐NiO exhibited significantly higher noble‐metal‐mass normalized HER activity compared to Pt_NC_‐P‐NiO and Pt/C, particularly in alkaline media, demonstrating superior Pt utilization and exceptional economic efficiency, thereby substantially reducing the HER mass activity gap between acidic and alkaline conditions (Figure S21c, Supporting Information).

The Pt_NC_‐D‐NiO also exhibited superior intrinsic HER activity in both alkaline and acidic media compared to Pt_NC_‐P‐NiO and Pt/C, as evidenced by the ECSA‐normalized HER polarization curves, highlighting a significantly reduced intrinsic activity gap between acidic and alkaline conditions (Figure S22a–c, Supporting Information). The intrinsic Tafel plots derived from the ECSA‐normalized HER current density further revealed that Pt_NC_‐D‐NiO exhibited the lowest Tafel slope in alkaline HER among the tested catalysts and a comparable Tafel slope in acidic media, indicating intrinsically faster alkaline HER kinetics due to the facilitated Volmer step (Figure S22d–f, Supporting Information). To quantitatively assess the acid–alkaline HER gap, we employed the activity ratio (*η*_alkaline_/*η*_acid_; geometric activity_acid_/geometric activity_alkaline_; mass activity_acid_/mass activity_alkaline_; intrinsic activity_acid_/intrinsic activity_alkaline_) and Tafel slope ratio (intrinsic Tafel slope_alkaline_/intrinsic Tafel slope_acid_) as HER gap factors (Figure 4b). A gap factor closer to 1 indicates a smaller activity discrepancy. The Pt_NC_‐D‐NiO exhibited significantly lower gap factors across all metrics compared to Pt/C and Pt_NC_‐P‐NiO, clearly demonstrating a reduced HER activity gap between acidic and alkaline conditions, attributed to the integration of Pt_NC_ on oxygen‐defect‐rich NiO nanowires.

### 2.4 Operando Characterizations of the Structure and Mechanistic Investigations

**Figure 5:**
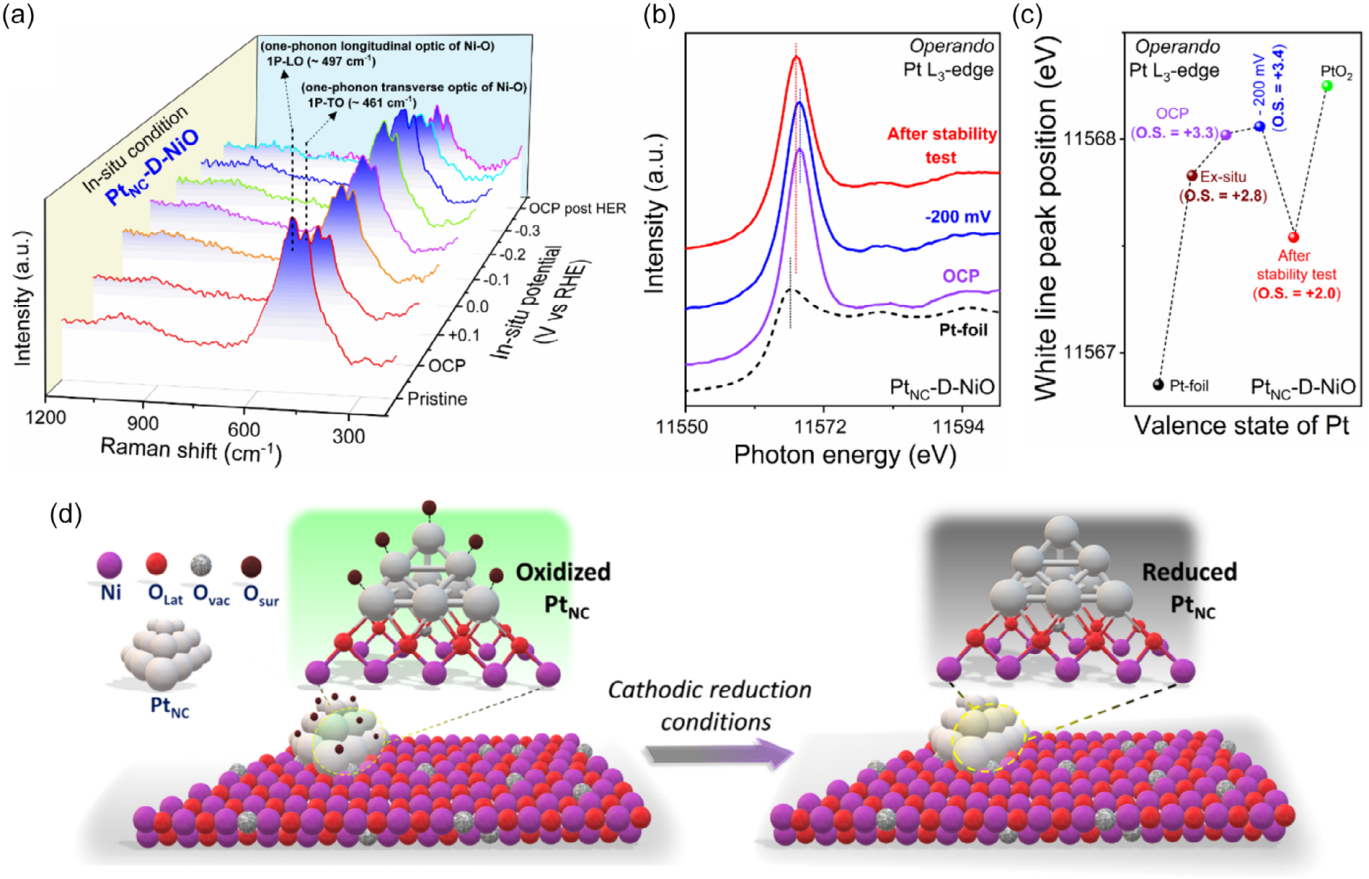
In situ Raman spectroscopy and operando XAS characterizations. a) In situ Raman spectroscopy measurements for Pt_NC_‐D‐NiO recorded during alkaline HER from +0.1 to −0.3 V versus RHE. b) Experimental Pt‐L_3_ edge operando XANES spectra of Pt_NC_‐D‐NiO at OCP, −200 mV overpotential and poststability test, compared with a Pt foil reference. c) Evolution of the Pt‐L_3_ edge maximums under applied potential, highlighting its correlation with the Pt oxidation state in Pt_NC_‐D‐NiO. d) Schematic illustration of the reduction of Pt_NC_ within Pt_NC_‐D‐NiO under the cathodic reduction conditions.

To gain deeper insights into the real‐time structural evolution of Pt_NC_‐D‐NiO under working conditions and to elucidate the atomic‐scale hydrogen spillover mechanism during alkaline HER, we employed a combination of in situ Raman spectroscopy, operando XAS, operando electrochemical impedance spectroscopy (EIS) measurements, and kinetic isotope effects (KIE) test in H_2_O and D_2_O electrolytes. In situ Raman measurement conducted during alkaline HER (**Figure** **5**a) revealed that the characteristics bands of Pt_NC_‐D‐NiO (1P‐TO ≈ 461 and 1P‐LO ≈ 497 cm^−1^, originated from the D‐NiO support) remained unchanged, indicating that the structural integrity of Pt_NC_‐D‐NiO was well preserved under alkaline HER conditions. Furthermore, operando Pt L_3_‐edge XAS analysis was performed to monitor potential‐dependent dynamic changes in the electronic structure of Pt_NC_ under alkaline HER operating conditions (Figure 5b,c). The white‐line peak position in the Pt L_3_‐edge XANES spectra at open‐circuit potential (OCP) indicated a Pt valence state of +3.3, slightly higher than that of the ex situ sample (+2.8), likely due to surface oxidation under harsh alkaline conditions. Upon applying a cathodic HER overpotential of −200 mV, the white‐line peak position remained largely unchanged, suggesting that H* adsorption at the HER working potential effectively prevented further oxidation of Pt_NC_ under alkaline conditions. Notably, after the long‐term stability test, the white‐line peak position exhibited a significant negative shift compared to OCP and ex situ conditions, indicating a reduction in the oxidation state of Pt_NC_. This suggests that under cathodic reduction conditions, the initially oxidized Pt_NC_ surface was significantly reduced, yielding a cleaner surface that facilitated efficient H* adsorption/desorption, ultimately enhancing overall HER kinetics, as schematically illustrated in Figure 5d.

**Figure 6:**
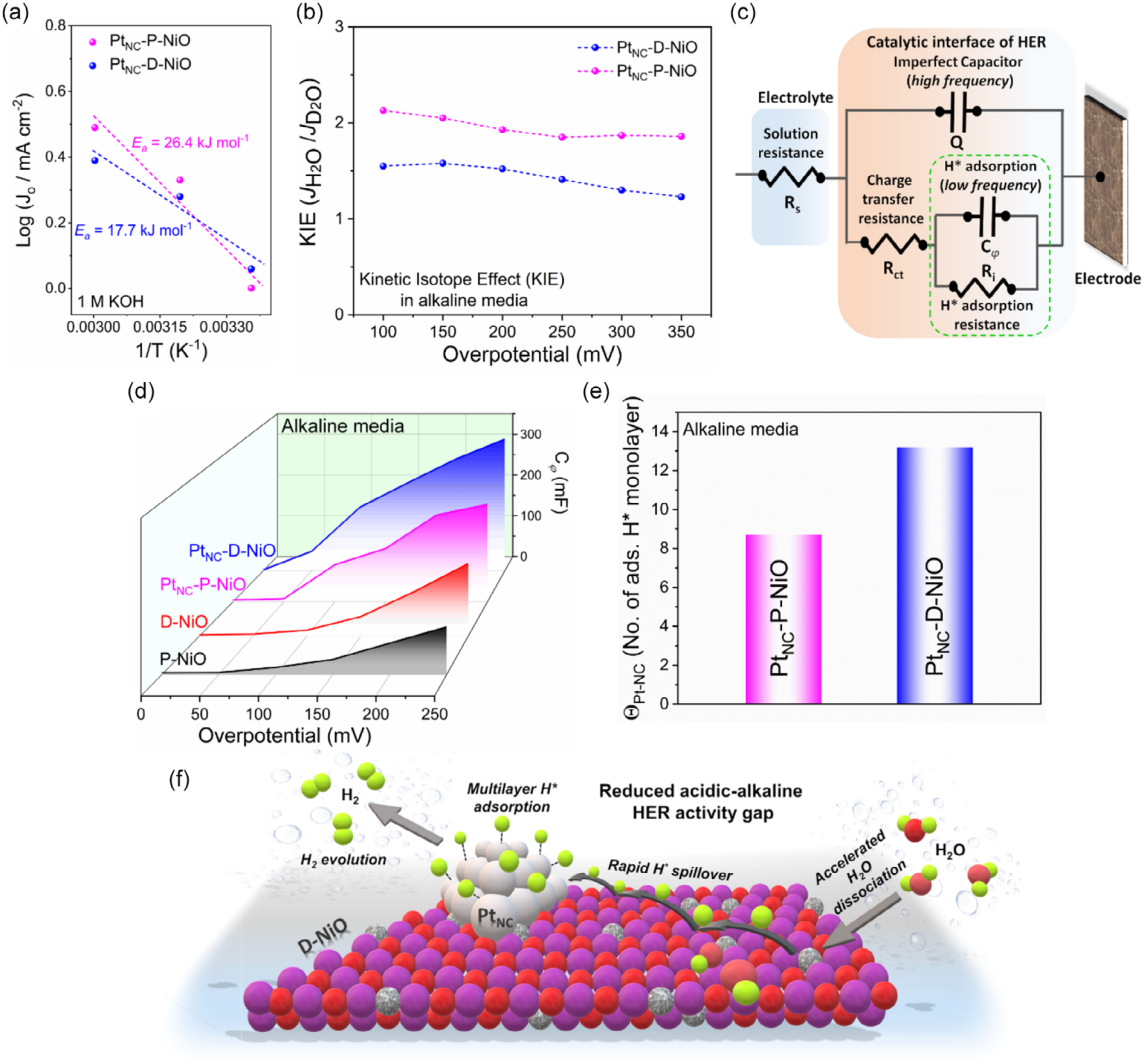
Experimental evidences for hydrogen spillover and mechanistic investigations. a) Arrhenius plot of the HER exchange current density, showing decreased activation energy (*E*_a_). b) Calculated KIE values under corresponding overpotentials for Pt_NC_‐D‐NiO and Pt_NC_‐P‐NiO alkaline medium. c) Equivalent circuit model used in the fitting of the impedance data. d) Plots of *C*_φ_ versus *η* for catalysts in alkaline medium. e) Comparison of Θ_Pt‐NC_ of Pt_NC_‐D‐NiO and Pt_NC_‐P‐NiO during HER in alkaline medium. f) Schematic illustrating the possible path of hydrogen spillover in alkaline environment.

To get clear insights into the catalytic mechanism, particularly in alkaline media, the activation energy (*E*_a_) of water dissociation was determined using temperature‐dependent HER polarization curves. The HER LSV curves recorded at different temperatures for both Pt_NC_‐D‐NiO and Pt_NC_‐P‐NiO in alkaline media showed a progressive decrease in onset potential and an increase in HER current with rising temperature, based on which the Arrhenius plots were constructed (Figure S23a,b, Supporting Information). According to the Arrhenius equation, *E*_a_ can be estimated from the slope of 1/*T* and log(*j*_o_), where *j*_o_ represents the exchange current density (**Figure** **6**a and Figure S23c,d, Supporting Information). The Pt_NC_‐D‐NiO exhibited an *E*_a_ of 17.7 kJ mol^−1^, which is ≈33% lower than that of Pt_NC_‐P‐NiO (26.4 kJ mol^−1^), indicating a significantly enhanced catalytic effect in alkaline media. To further confirm the water dissociation ability, hydrazine oxidation experiments were conducted, as the oxidation onset potential and current density serve as indicators of water dissociation efficiency (lower onset potential and higher current density correspond to improved water dissociation capability). As shown in Figure S24 (Supporting Information), the Pt_NC_ integrated onto defect rich NiO (Pt_NC_‐D‐NiO) exhibited a lower hydrazine oxidation onset potential and a higher current density compared to Pt_NC_‐P‐NiO, corroborating its superior water dissociation ability.

To further elucidate H* transfer kinetics, KIE experiments were performed for Pt_NC_‐P‐NiO and Pt_NC_‐D‐NiO in H_2_O and D_2_O electrolytes (Figure S25, Supporting Information and Figure 6b). The KIE values for both catalysts were greater than 1, confirming that H* transfer is indeed involved in the rate‐limiting step of the alkaline HER process.^[^ ^10^ ^]^ Notably, Pt_NC_‐D‐NiO exhibited a lower KIE value compared to Pt_NC_‐P‐NiO, indicating more facile H—OH bond dissociation (enhanced water dissociation) and improved H* transfer kinetics, likely facilitated by oxygen vacancy‐mediated hydrogen spillover.^[^ ^10^ ^]^

Additionally, operando EIS measurements were conducted to assess the adsorption strength of H* species and track H* transfer dynamics around active sites during the HER.^[^ ^32^, ^33^ ^]^ The operando EIS measurements were performed at various applied HER overpotentials (*η*) and the EIS plots were simulated using a double‐parallel equivalent circuit model, where *C*_φ_ represents the H* adsorption pseudocapacitance and *R*_i_ denotes the H* adsorption resistance (Figure S26, Supporting Information, Figure 6c, and Table S4, Supporting Information). The lower *R*_i_ values for Pt_NC_‐D‐NiO and Pt_NC_‐P‐NiO compared to their respective D‐NiO and P‐NiO supports suggest that Pt_NC_ integration facilitates H* adsorption. To further quantify H* adsorption capacity, the H* adsorption charge (*Q*_H*_) was determined by integrating the *C*_φ_ versus *η* profile, providing insights into the amount of H* adsorbed on the catalyst surface during HER (Figure 6d).^[^ ^33^ ^]^ Over the potential range of 0 to 250 mV, Pt_NC_‐D‐NiO exhibited the highest *Q*_H*_, indicating significantly enhanced H* coverage and favorable H* adsorption, attributed to improved water dissociation and the H* spillover process. Furthermore, assuming that the H* adsorption charge for a monolayer is equivalent to that of a Pt(111) surface (*Q*_standard_ = 210 μC cm^−2^), the apparent H* coverage of Pt_NC_ (Θ_Pt‐NC_) for Pt_NC_‐D‐NiO and Pt_NC_‐P‐NiO was quantified (Figure 6e, Discussion S1, and S2, Supporting Information). The higher Θ_Pt‐NC_ observed for Pt_NC_‐D‐NiO suggests a greater H* concentration on Pt_NC_ sites supported on D‐NiO, thereby enhancing alkaline HER kinetics. As schematically illustrated in Figure 6f, the integration of Pt_NC_ on the D‐NiO support enhances HER kinetics in alkaline media through the synergistic effect of improved water dissociation and efficient hydrogen spillover, mediated by oxygen‐deficient sites. This synergism effectively accelerates the reaction kinetics and mitigates the activity disparity between acidic and alkaline HER.

## 3 Conclusion

In summary, we developed a facile strategy to integrate ultrasmall Pt_NCs_ onto oxygen‐defect‐rich NiO nanowires, forming a binary‐component electrocatalyst that achieves synergistic water dissociation and hydrogen spillover for highly efficient HER in both alkaline and acidic media. Through a combination of ex situ and operando spectroscopic and electrochemical analyses, we addressed the mechanistic gap by experimentally uncovering how oxygen vacancies mediate hydrogen spillover. Specifically, oxygen‐defect sites on NiO facilitate the initial water dissociation and H generation, while strong Pt—O_support_ coordination enables rapid interfacial H migration to the Pt_NC_ surface, completing the spillover process. Operando EIS, KIE measurements, and hydrazine oxidation experiments consistently confirm that the presence of surface oxygen vacancies markedly lowers the water dissociation barrier and enhances interfacial charge transfer kinetics. This defect‐driven spillover mechanism effectively bridges the intrinsic HER activity gap between alkaline and acidic conditions. Consequently, Pt_NC_‐D‐NiO delivers superior intrinsic and mass‐normalized HER activity, coupled with exceptional long‐term durability exceeding 150 h without significant degradation. Overall, this study provides direct mechanistic evidence for vacancy‐facilitated hydrogen spillover and establishes a general design principle for engineering metal–oxide interfaces toward practical, cost‐effective, and durable electrocatalysts for hydrogen production.

## 4 Experimental Section

4.1

4.1.1

##### Materials

Nickel(II) nitrate hexahydrate (Ni(NO_3_)_2_·6H_2_O; Sigma–Aldrich, ≥99%), hexachloroplatinic acid hexahydrate (H_2_PtCl_6_.6H_2_O; Sigma–Aldrich, ≥99.9%), potassium hydroxide (KOH; Sigma–Aldrich, ≥85%), sulfuric acid (H_2_SO_4_; Sigma–Aldrich, ≥99.99%), ethanol (C_2_H_5_OH; Sigma–Aldrich, ≥99.9%), commercial Pt/C 20 wt% (<5 nm; Sigma–Aldrich, 100%), CFP: carbon fiber paper (Toray TGP‐H‐60 non‐PTFE coated), and the Nafion perfluorinated resin solution (5 wt%, Sigma–Aldrich) were used without further purification.

##### Synthesis of P‐NiO and D‐NiO Supports

The P‐NiO and D‐NiO nanowires were synthesized via the nanocasting method using SBA‐15 silica as a hard template, which was aged at 100 °C.^[^ ^34^, ^35^ ^]^ The precursor solutions were prepared by dissolving 1.94 g of Ni(NO_3_)_2_·6H_2_O in 7 mL of ethanol, followed by a two‐step impregnation process using half of the stock solution in each cycle. The impregnated composite was then subjected to calcination at 450 °C for 4 h, under air to obtain P‐NiO and under an argon atmosphere to obtain D‐NiO. Finally, the SBA‐15 template was removed by etching with hot 2 m NaOH solution, yielding the assembly of nanowire powder samples.

##### Material Characterization

The structural and crystallographic properties, including crystal orientations of all catalysts, were analyzed using XRD on a Stoe theta/theta diffractometer operating in reflection mode with Cu Kα_1,2_ radiation X‐ray source (*λ*: 1.5406 Å). TEM images were acquired using a Thermo Scientific Talos F200X (S)TEM microscope equipped with a high brightness field emission gun (X‐FEG) and operated at an acceleration voltage of 200 kV. Elemental analysis via EDS was performed using an EDAX Octane T Ultra W 200 mm^2^ silicon drift detector (SDD). ICP‐OES measurements were conducted using a SPECTROGREEN instrument. Dark‐field imaging was carried out with a Hitachi HD‐2700 Cs‐corrected scanning transmission electron microscope equipped with a cold field‐emission gun and operated at 200 keV. XPS analysis was performed using a VG ESCALAB 220i‐XL spectrometer with a monochromatic Al Kα X‐ray source (1486.6 eV), operating at 200 W and 15 kV. The analysis chamber maintained a base pressure of 5 × 10^−10^ mbar. The C 1*s* peak of adventitious carbon at 284.5 eV was used as a reference for binding energy calibration to correct for surface charging effects.

##### XAS Measurements: Ex Situ Measurements

Ni K‐edge and Pt L_3_‐edge XAS measurements for Pt_NC_‐P‐NiO and Pt_NC_‐D‐NiO were conducted in fluorescence mode, while reference samples, including Ni(0), Pt(0), and PtO_2_, were analyzed in transmission mode. Spectra were acquired at the BL10C beamline of the Pohang Light Source (PLS‐II, Korea) under top‐up mode operation, maintaining a ring current of 250 mA at 3.0 GeV. Powder samples were prepared by homogenizing the catalyst powders with cellulose, followed by pelletization (*φ* = 13 mm). The pellets were then mounted in a sample holder and sealed with a 30 μm Kapton tape. A monochromatic X‐ray beam was generated using a liquid‐nitrogen‐cooled Si(111) double‐crystal monochromator (Bruker ASC), sourced from a high‐intensity multipole wiggler. X‐ray absorption data were recorded in fluorescence mode using a seven‐channel SDD (Rayspec Ltd.), offering high sensitivity for low‐concentration elements. To suppress higher‐order harmonic contamination, the incident X‐ray intensity was reduced by ≈30% through detuning. Energy calibration was performed in real time by positioning the corresponding metallic reference foil in front of the third ion chamber, with the inflection point of the metal foil used as the calibration reference. Data processing and normalization were carried out using the Fastosh software.

##### XAS Measurements: Operando XAS

Operando Pt L_3_‐edge XAS measurements were performed to investigate the electronic structure of Pt_NC_‐D‐NiO under reaction conditions. The experiments were conducted at the SAMBA beamline of the SOLEIL synchrotron, utilizing a 450 mA electron beam (beam size: 4 mm × 0.5 mm). The incident energy was selected using a Si(200) double‐crystal monochromator, and fluorescence spectra were recorded with a 36‐element germanium detector. The Pt_NC_‐D‐NiO electrodes were positioned in a spectro‐electrochemical flow cell, secured with a custom‐designed plate. A continuous flow of 1 m KOH electrolyte was maintained via a membrane pump (Fluigent) regulated by a vacuum pressure‐based controller to ensure solution refreshment and pH stability. Electrochemical measurements were performed using a SP‐300 potentiostat (Biologic). Prior to controlled potential electrolysis at an overpotential of −200 mV, five cyclic voltammetry (CV) cycles were conducted to stabilize the electrode and determine the applied potential. All measurements were conducted at room temperature, with no observable radiation‐induced damage. The incident energy was calibrated against the inflection point of a Pt foil, and the acquired spectra were processed and normalized using Fastosh software.

##### Electrochemical Measurements

Electrochemical measurements were performed using a VSP‐300 BioLogic potentiostat in a conventional three‐electrode configuration with 1 m KOH and 0.5 m H_2_SO_4_ as the electrolyte. A catalyst ink‐coated carbon fiber paper served as the working electrode, while a Hg/HgO (1 m KOH) or Ag/AgCl (3 m KCl) electrode functioned as the reference, and a Pt mesh was used as the counter electrode. The reference electrode potential was calibrated in a H_2_‐saturated electrolyte, and all potentials were converted to the RHE scale using the equation:
(1)

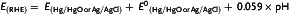




For catalyst ink preparation, 5 mg of catalyst powder was dispersed in 500 μL of ethanol containing 20 μL of 5% Nafion, followed by sonication for 60 min to achieve a homogeneous dispersion. A defined volume of the ink was then drop‐cast onto carbon fiber paper(CFP) (loading: 1 mg cm^−2^) and dried under ambient conditions. Prior to electrochemical testing, the electrodes were conditioned via CV at a scan rate of 100 mV s^−1^ in an argon‐purged electrolyte. LSV was performed at a slow scan rate of 5 mV s^−1^ to minimize capacitive effects. Nyquist plots were recorded via EIS in the Faradaic region to determine the solution resistance (*R*_s_) and charge transfer resistance (*R*_CT_). The double‐layer capacitance (*C*_dl_) was estimated from CV measurements at different scan rates (10–30 mV s^−1^) in the non‐Faradaic region, and the ECSA was derived using a specific capacitance of 0.06 mF cm^−2^. Long‐term stability was assessed via chronopotentiometry at a constant current density of −50 mA cm^−2^. Unless stated otherwise, all potentials were corrected for *iR* drop using the equation:

(2) $$E_{\text{corrected}}=\;E_{\text{(RHE)}}–\;iR$$

##### In Situ Raman Spectroscopy Measurement

In situ Raman measurements were performed using a customized electrochemical flow cell integrated with an InVia Renishaw Raman microscope. The system employed a 532 nm laser excitation source with an 1800 L mm^−1^ grating and a 50× objective lens. The catalyst ink was drop‐cast onto roughened Au foil, which served as the working electrode, while a Pt wire and a Hg/HgO electrode were used as the counter and reference electrodes, respectively. A 0.1 m KOH electrolyte (pH 13) was used to facilitate the HER. During measurements, the electrolyte was continuously circulated at a controlled flow rate of 5–8 mL min^−1^ using a peristaltic pump. In situ Raman spectra were collected over ten consecutive scans with a 10 s exposure time per scan at a laser power of 0.5 mW. The electrochemical measurements were conducted in chronoamperometric mode, with the applied potential held for 3 min at each step, ranging from +0.1 to −0.3 V versus RHE.

## Supporting Information

Supporting Information is available from the Wiley Online Library or from the author.

## Conflict of Interest

The authors declare no conflict of interest.

## Supporting information

Supplementary Material

## Data Availability

The data that support the findings of this study are available from the corresponding author upon reasonable request.

## References

[1] M. Yu , E. Budiyanto , H. Tüysüz , Angew. Chem., Int. Ed. 2022, 61, 202103824. 10.1002/anie.202103824 PMC9291824 34138511

[2] A. Kumar , V. Q. Bui , J. Lee , L. L. Wang , A. R. Jadhav , X. H. Liu , X. D. Shao , Y. Liu , J. M. Yu , Y. Hwang , H. T. D. Bui , S. Ajmal , M. G. Kim , S. G. Kim , G. S. Park , Y. Kawazoe , H. Lee , Nat. Commun. 2021, 12, 6766. 34799571 10.1038/s41467-021-27145-3 PMC8604929

[3] Y. Zheng , Y. Jiao , A. Vasileff , S.‐Z. Qiao , Angew. Chem., Int. Ed. 2018, 57, 7568. 10.1002/anie.201710556 29194903

[4] Y. Liu , J. Ding , F. Li , X. Su , Q. Zhang , G. Guan , F. Hu , J. Zhang , Q. Wang , Y. Jiang , B. Liu , H. B. Yang , Adv. Mater. 2023, 35, e2207114. 36205652 10.1002/adma.202207114

[5] A. Kumar , V. Q. Bui , J. Lee , A. R. Jadhav , Y. Hwang , M. G. Kim , Y. Kawazoe , H. Lee , ACS Energy Lett. 2021, 6, 354.

[6] S. Shrestha , Y. Liu , W. E. Mustain , Catal. Rev. 2011, 53, 256.

[7] J. Zhang , L. Zhang , J. Liu , C. Zhong , Y. Tu , P. Li , L. Du , S. Chen , Z. Cui , Nat. Commun. 2022, 13, 5497. 36127343 10.1038/s41467-022-33216-w PMC9489878

[8] J. Wang , S.‐J. Kim , J. Liu , Y. Gao , S. Choi , J. Han , H. Shin , S. Jo , J. Kim , F. Ciucci , H. Kim , Q. Li , W. Yang , X. Long , S. Yang , S.‐P. Cho , K. H. Chae , M. G. Kim , H. Kim , J. Lim , Nat. Catal. 2021, 4, 212.

[9] T. F. Jaramillo , K. P. Jørgensen , J. Bonde , J. H. Nielsen , S. Horch , I. Chorkendorff , Science 2007, 317, 100. 17615351 10.1126/science.1141483

[10] H. Q. Fu , M. Zhou , P. F. Liu , P. Liu , H. Yin , K. Z. Sun , H. G. Yang , M. Al‐Mamun , P. Hu , H. F. Wang , H. Zhao , J. Am. Chem. Soc. 2022, 144, 6028. 35302356 10.1021/jacs.2c01094

[11] L. Zhu , H. Lin , Y. Li , F. Liao , Y. Lifshitz , M. Sheng , S.‐T. Lee , M. Shao , Nat. Commun. 2016, 7, 12272. 27447292 10.1038/ncomms12272 PMC4961846

[12] W. Karim , C. Spreafico , A. Kleibert , J. Gobrecht , J. VandeVondele , Y. Ekinci , J. A. van Bokhoven , Nature 2017, 541, 68. 28054605 10.1038/nature20782

[13] Y. Liu , H. T. Bui , A. R. Jadhav , T. Yang , S. Saqlain , Y. Luo , J. Yu , A. Kumar , H. Wang , L. Wang , Adv. Funct. Mater. 2021, 31, 2010718.

[14] A. Kumar , J. Lee , M. G. Kim , B. Debnath , X. H. Liu , Y. Hwang , Y. Wang , X. D. Shao , A. R. Jadhav , Y. Liu , H. Tüysüz , H. Lee , ACS Nano 2022, 16, 15297. 36099061 10.1021/acsnano.2c06747

[15] J. Lee , A. Kumar , M. G. Kim , T. Yang , X. Shao , X. Liu , Y. Liu , Y. Hong , A. R. Jadhav , M. Liang , N. Q. Tran , H. Lee , ACS Energy Lett. 2021, 6, 4299.

[16] Z. W. Wei , H. J. Wang , C. Zhang , K. Xu , X. L. Lu , T. B. Lu , Angew. Chem., Int. Ed. 2021, 60, 16622. 10.1002/anie.202104856 34002464

[17] S. Zhou , H. Jang , Q. Qin , L. Hou , M. G. Kim , S. Liu , X. Liu , J. Cho , Angew. Chem., Int. Ed. 2022, 61, e202212196. 10.1002/anie.202212196 36164268

[18] J. G. Highfield , E. Claude , K. Oguro , Electrochim. Acta 1999, 44, 2805.

[19] Y. Cheng , S. Lu , F. Liao , L. Liu , Y. Li , M. Shao , Adv. Funct. Mater. 2017, 27, 1700359.

[20] H. Tian , X. Cui , L. Zeng , L. Su , Y. Song , J. Shi , J. Mater. Chem. A 2019, 7, 6285.

[21] E. Budiyanto , S. Salamon , Y. Wang , H. Wende , H. Tüysüz , JACS Au 2022, 2, 697. 35373196 10.1021/jacsau.1c00561 PMC8970005

[22] A. Kumar , M. Gil‐Sepulcre , J. P. Fandré , O. Rüdiger , M. G. Kim , S. DeBeer , H. Tüysüz , J. Am. Chem. Soc. 2024, 146, 32953. 39378366 10.1021/jacs.4c08847 PMC11622227

[23] S. Yao , H. Wei , Y. Zhang , X. Zhang , Y. Wang , J. Liu , H. H. Tan , T. Xie , Y. Wu , Catal. Sci. Technol. 2021, 11, 264.

[24] K. Zhang , J. Liu , L. Wang , B. Jin , X. Yang , S. Zhang , J. H. Park , J. Am. Chem. Soc. 2020, 142, 8641. 32160742 10.1021/jacs.9b13410

[25] N. A. Sagui , P. Ström , T. Edvinsson , İ. B. Pehlivan , ACS Catal. 2022, 12, 6506. 35692248 10.1021/acscatal.2c00577 PMC9171717

[26] K. Tang , W. Liu , J. Li , J. Guo , J. Zhang , S. Wang , S. Niu , Y. Yang , ACS Appl. Mater. Interfaces 2015, 7, 26839. 26573213 10.1021/acsami.5b09110

[27] K. Wang , S. Wang , K. S. Hui , J. Li , C. Zha , D. A. Dinh , Z. Shao , B. Yan , Z. Tang , K. N. Hui , Adv. Funct. Mater. 2022, 33, 2211273.

[28] A. Kumar , M. Gil‐Sepulcre , J. Lee , V. Q. Bui , Y. Wang , O. Rüdiger , M. G. Kim , S. DeBeer , H. Tüysüz , Adv. Mater. 2024, 36, 2401648. 10.1002/adma.202401648 39318088

[29] H. Jin , C. Guo , X. Liu , J. Liu , A. Vasileff , Y. Jiao , Y. Zheng , S.‐Z. Qiao , Chem. Rev. 2018, 118, 6337. 29552883 10.1021/acs.chemrev.7b00689

[30] Z. Chen , D. Cummins , B. N. Reinecke , E. Clark , M. K. Sunkara , T. F. Jaramillo , Nano Lett. 2011, 11, 4168. 21894935 10.1021/nl2020476

[31] Y. Liu , Q. Li , R. Si , G. D. Li , W. Li , D. P. Liu , D. Wang , L. Sun , Y. Zhang , X. Zou , Adv. Mater. 2017, 29, 1606200. 10.1002/adma.201606200 28128868

[32] J. Chen , C. Chen , M. Qin , B. Li , B. Lin , Q. Mao , H. Yang , B. Liu , Y. Wang , Nat. Commun. 2022, 13, 5382. 36104336 10.1038/s41467-022-33007-3 PMC9474501

[33] Y. Liu , X. Liu , A. R. Jadhav , T. Yang , Y. Hwang , H. Wang , L. Wang , Y. Luo , A. Kumar , J. Lee , H. T. D. Bui , M. Gyu Kim , H. Lee , Angew. Chem., Int. Ed. 2022, 61, e202114160. 10.1002/anie.202114160 34964231

[34] E. Budiyanto , M. Yu , M. Chen , S. DeBeer , O. Ruediger , H. Tüysüz , ACS Appl. Energy Mater. 2020, 3, 8583.

[35] X. Deng , K. Chen , H. Tüysüz , Chem. Mater. 2017, 29, 40.

