# Supplementary material for: Enhanced Proton Spillover at Pt‐Cluster/NiO Interface Reduces the Acidic–Alkaline Hydrogen Evolution Activity Gap
Source: Small Sci. 2026 Jan 28;6(1):e202500627. doi: 10.1002/smsc.202500627 (PMC12853402; doi:10.1002/smsc.202500627)
Supplement: Supplementary file 1 — Supplementary Material [file SMSC-6-e202500627-s001.pdf]

Supporting information

Enhanced Proton Spillover at Pt-cluster/NiO Interface Reduces the Acidic-Alkaline Hydrogen Evolution Activity Gap

*Ashwani Kumar, Jinsun Lee, Min Gyu Kim, Harun Tüysüz**

Dr. A. Kumar, Dr. J. Lee, Priv.-Doz. Dr. Harun Tüysüz
Department of Heterogeneous Catalysis, Max-Planck-Institut für Kohlenforschung, Kaiser-Wilhelm-Platz 1, 45470, Mülheim an der Ruhr (Germany)
E-mail: tueysuez@kofo.mpg.de

Dr. M. G. Kim
Beamline Research Division, Pohang Accelerator Laboratory (PAL), Pohang 790-784, South Korea

Prof. Dr. habil. H. Tüysüz
Catalysis and Energy Materials, IMDEA Materials Institute, Calle Eric Kandel 2, Getafe, Madrid, 28906, Spain

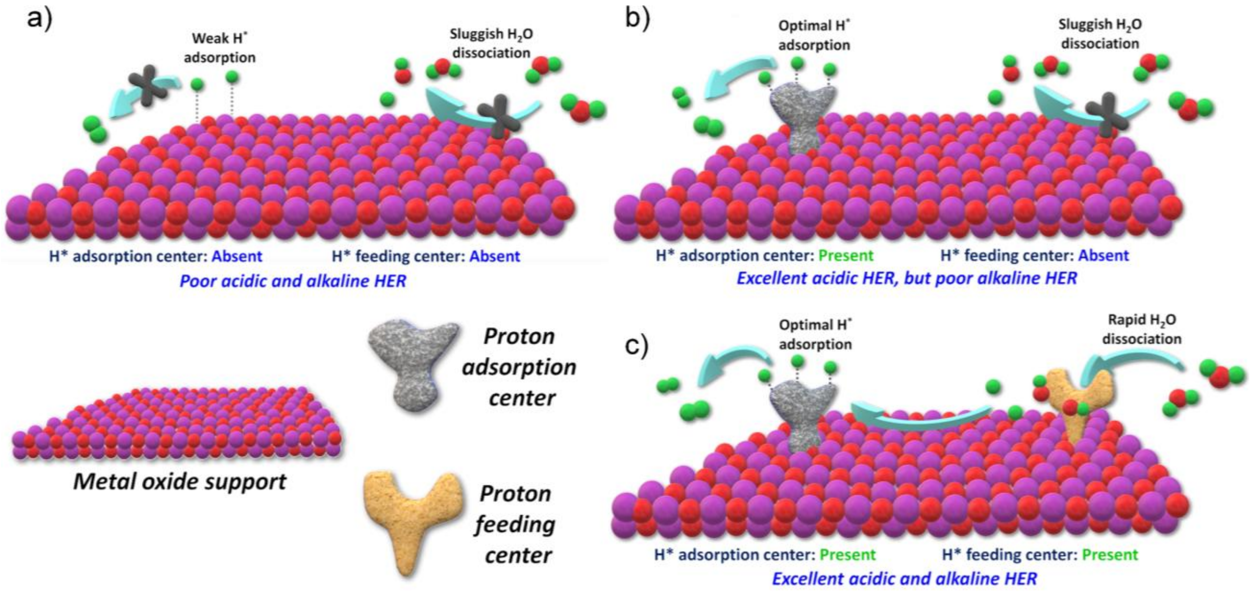

Figure S1. Schematic illustration for the acidic and alkaline HER on (a) metal oxide, (b) metal oxide modified with proton adsorption center and (c) metal oxide modified with proton adsorption and feeding center.

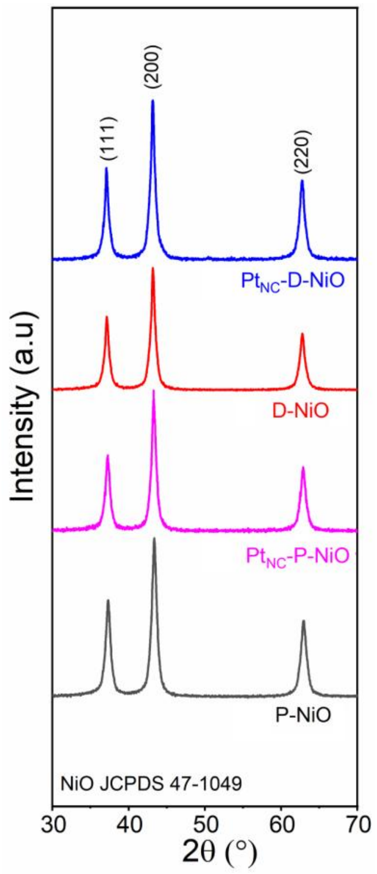

Figure S2. XRD patterns of P-NiO, D-NiO, Pt_{NC}-P-NiO, and Pt_{NC}-D-NiO.

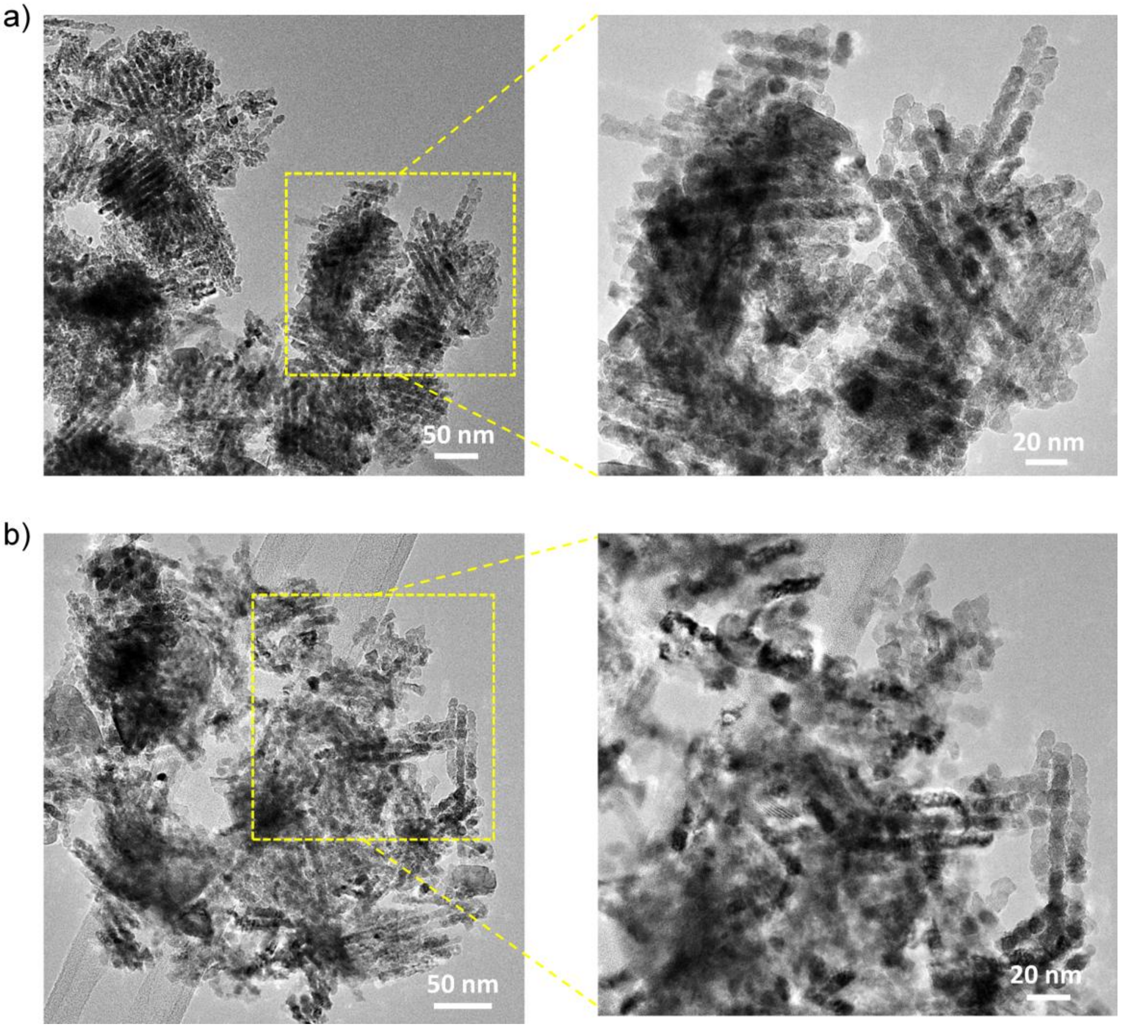

Figure S3. TEM images of (a) P-NiO, and (b) D-NiO.

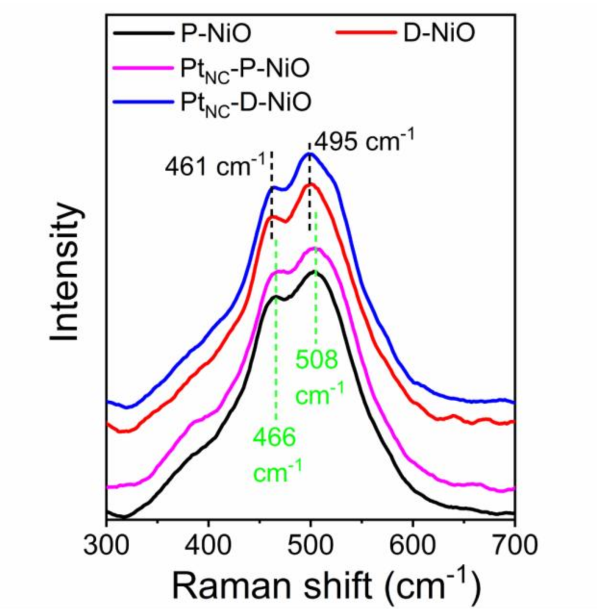

Figure S4. The Raman spectra of P-NiO, D-NiO, Pt_{NC}-P-NiO, and Pt_{NC}-D-NiO.

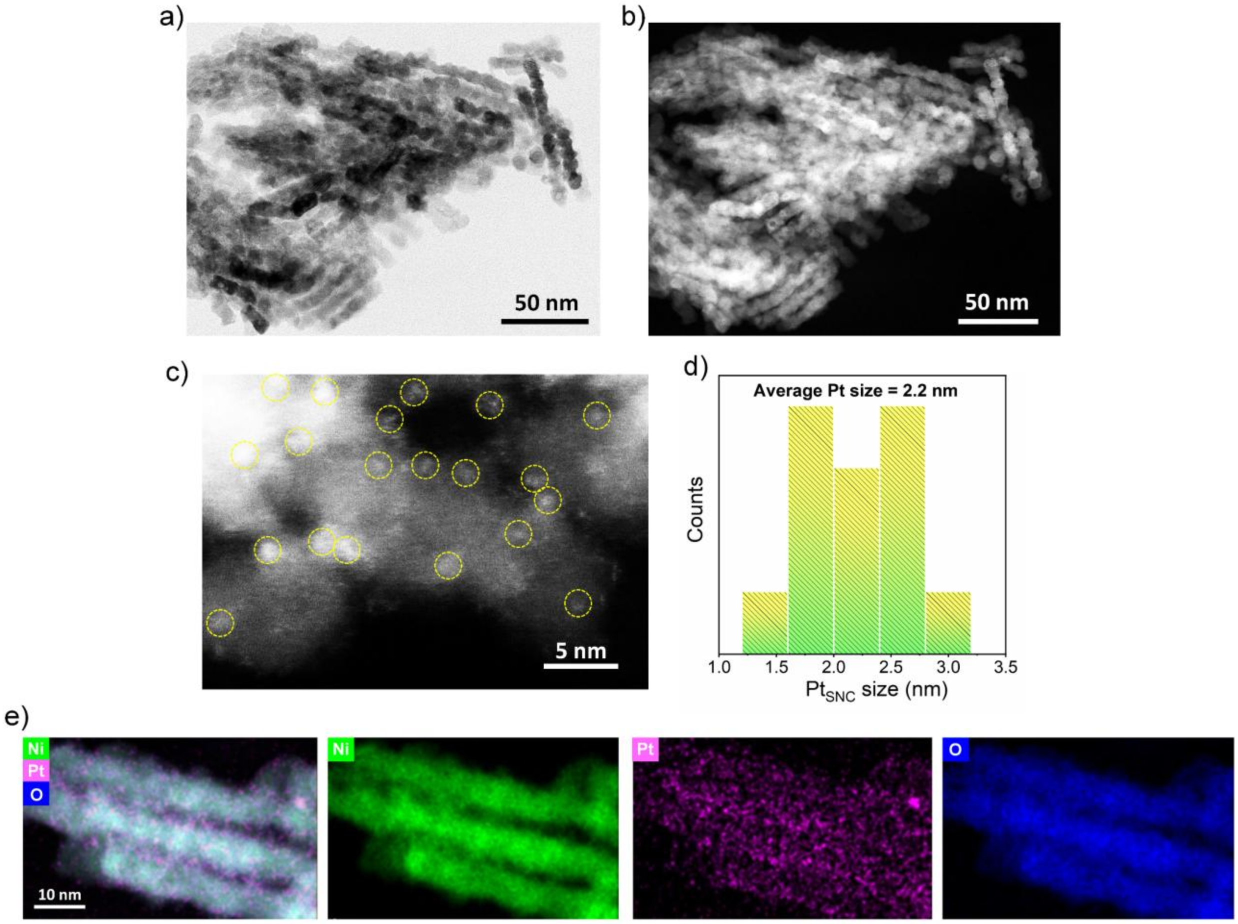

Figure S5. (a) TEM and (b) HAADF-STEM image of Pt_{NC}-D-NiO. (c) Magnified HAADF-STEM image of Pt_{NC}-D-NiO. (d) The size distribution of amorphous Pt nanoclusters within Pt_{NC}-D-NiO. (e) Corresponding elemental mapping images of Pt_{NC}-D-NiO.

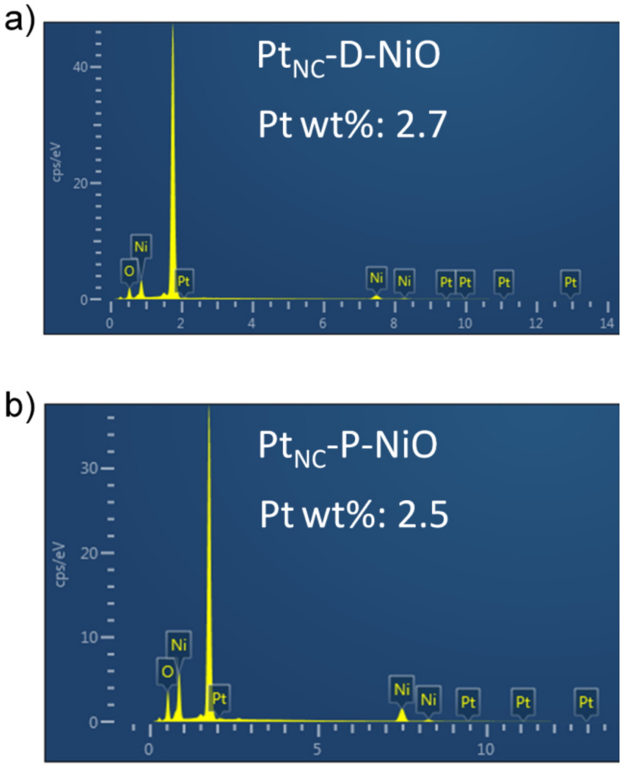

Figure S6. EDS pattern of (a) Pt_{NC}-D-NiO, and (b) Pt_{NC}-P-NiO.

Table S1. Weight % of metal loading obtained from EDS and ICP-OES analysis.

**Sample**	**ICP-OES (Pt wt%)**	**Average Pt (wt%) from EDS and ICP-OES**
Pt_{NC}-P-NiO	2.3	~ 2.4 ± 0.1
Pt_{NC}-D-NiO	2.0	~ 2.4 ± 0.3

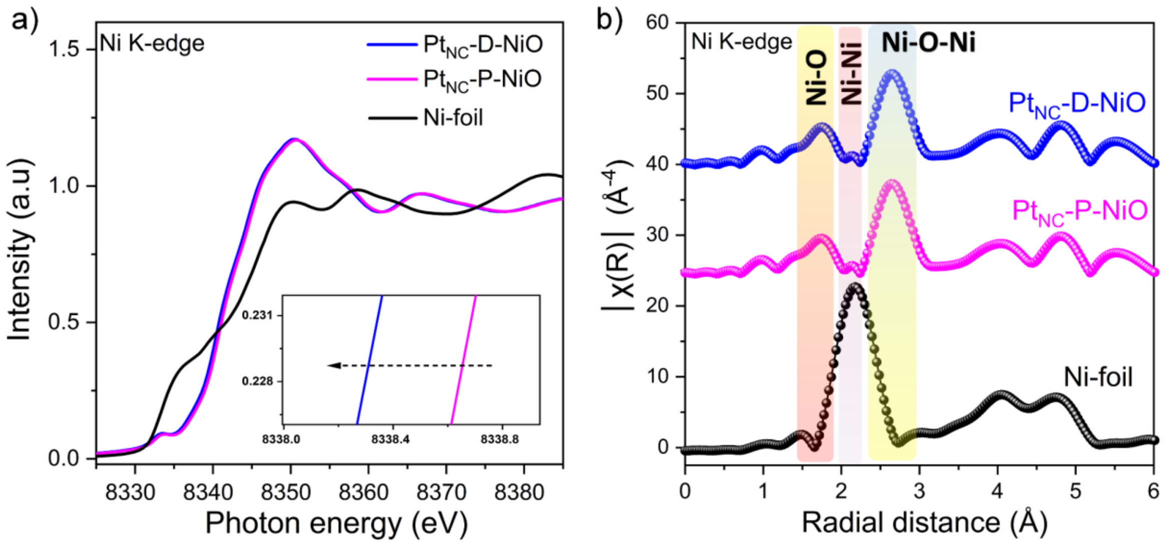

Figure S7. Experimental Ni K-edge (a) XANES and (b) FT-EXAFS spectra of Pt_{NC}-D-NiO and Pt_{NC}-P-NiO with Ni foil as reference sample.

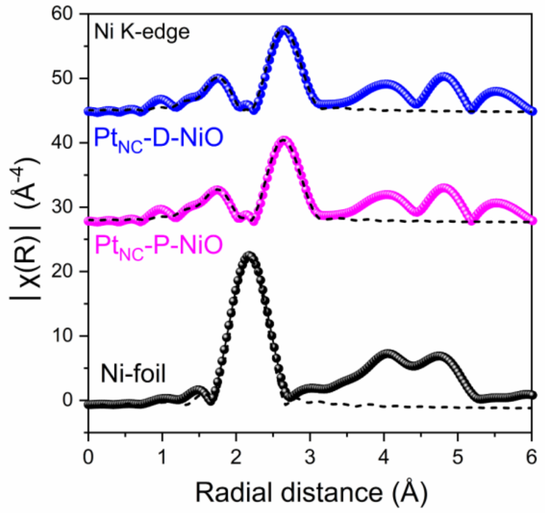

Figure S8. Experimental (solid spheres) and fitted (dashed line) Ni K-edge FT-EXAFS spectra of Pt_{NC}-D-NiO and Pt_{NC}-P-NiO with Ni foil as reference sample.

Table S2. Summary of the fitting parameters for Ni K-edge FT-EXAFS region.

Sample	Region	Path	CN	R, Å	$\sigma^2(10^{-3})$, Å^2	ΔE_0, eV	R-factor
**Ni foil**							
Ni K-edge	k = 3 - 12 Å^{-1}	Ni-Ni	12	2.48	6.1	7.3	0.007
	R = 1.7 - 2.72 Å						
**Pt_{NC}-P-NiO**							
Ni K-edge	k = 3 - 12 Å^{-1}	Ni-O	5.7	2.1	9.4	5.98	0.023
	R = 1.2 – 3.1 Å	Ni-Ni	9.8	2.94	6.9	5.98	
**Pt_{NC}-D-NiO**							
Ni K-edge	k = 3 - 12 Å^{-1}	Ni-O	4.6	2.1	6.2	5.8	0.015
	R = 1.2 – 3.1 Å	Ni-Ni	9.2	2.94	6.3	5.8	

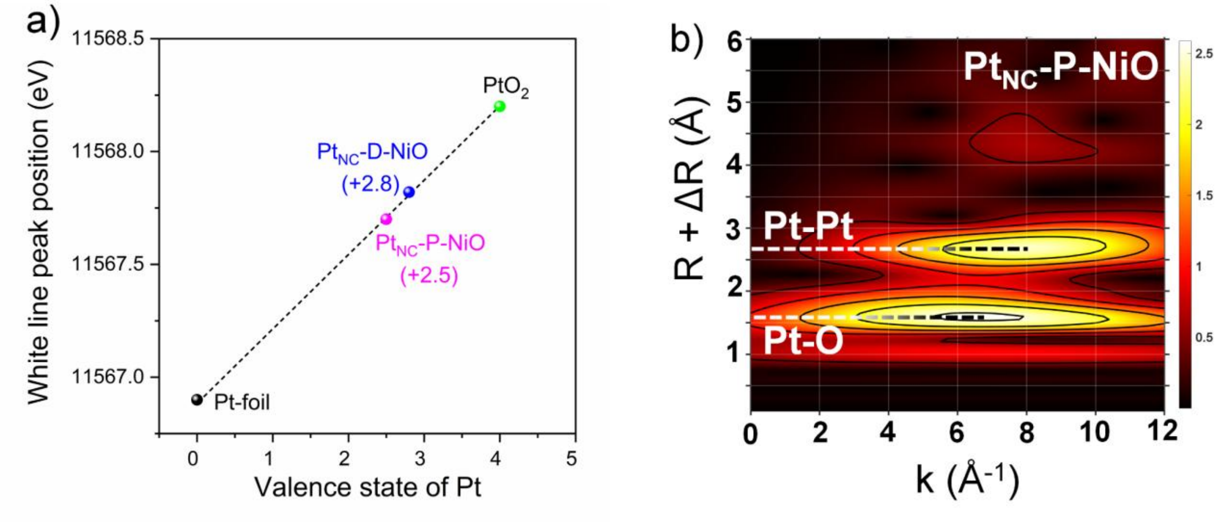

Figure S9. (a) Pt oxidation state analysis by corresponding Pt L_3-edge XANES white line peak position. (b) Wavelet transform EXAFS (WT-EXAFS) of Pt_{NC}-P-NiO.

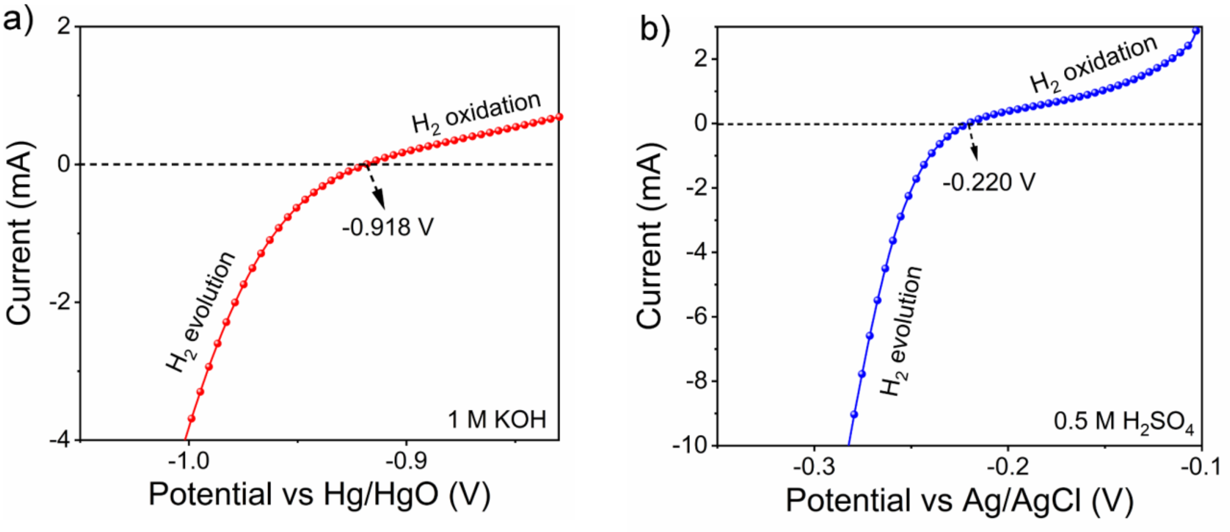

Figure S10. (a) Hg/HgO and (b) Ag/AgCl reference electrode calibration in H_2 saturated 1 M KOH and 0.5 M H_2SO_4, respectively.

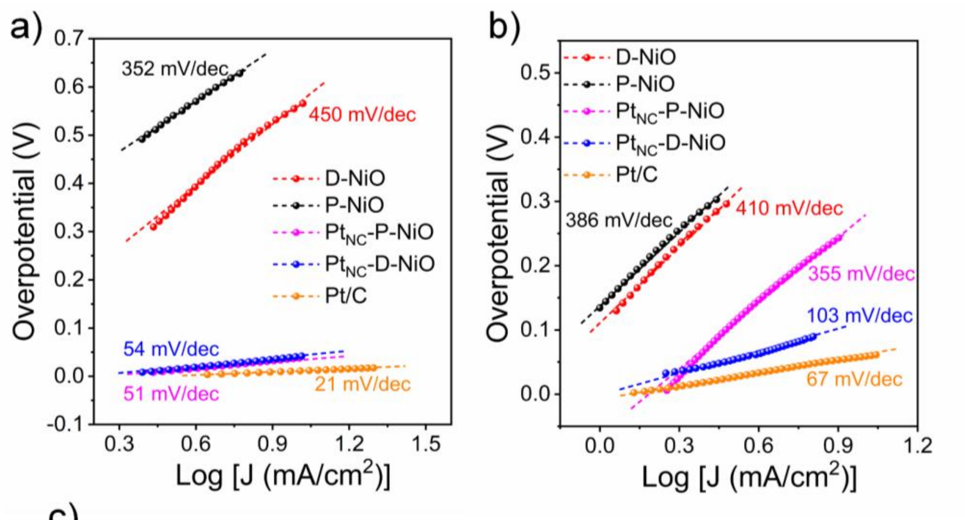

c)

	Acidic	Alkaline	Tafel slope
Overall	$* + 2H^+ + 2e^- \rightarrow H_2$	$* + 2H_2O + 2e^- \rightarrow H_2 + 2OH^-$	
Volmer	$* + H^+ + e^- \rightarrow H*$	$* + H_2O + e^- \rightarrow H* + OH^-$	$b = 2.3RT/\alpha F \approx 120$ mV/dec
Heyrovsky	$* + H^+ + e^- + H* \rightarrow H_2 + *$	$* + H_2O + e^- + H* \rightarrow H_2 + * + OH^-$	$b = 2.3RT/(1+\alpha)F \approx 40$ mV/dec
Tafel	$2H* \rightarrow H_2 + 2*$	$2H* \rightarrow H_2 + 2*$	$b = 2.3RT/2F \approx 30$ mV/dec

* denotes an active site

Figure S11. Corresponding Tafel plots in (a) acidic and (b) alkaline medium. (c) HER mechanism with elementary steps in acid and alkaline medium.

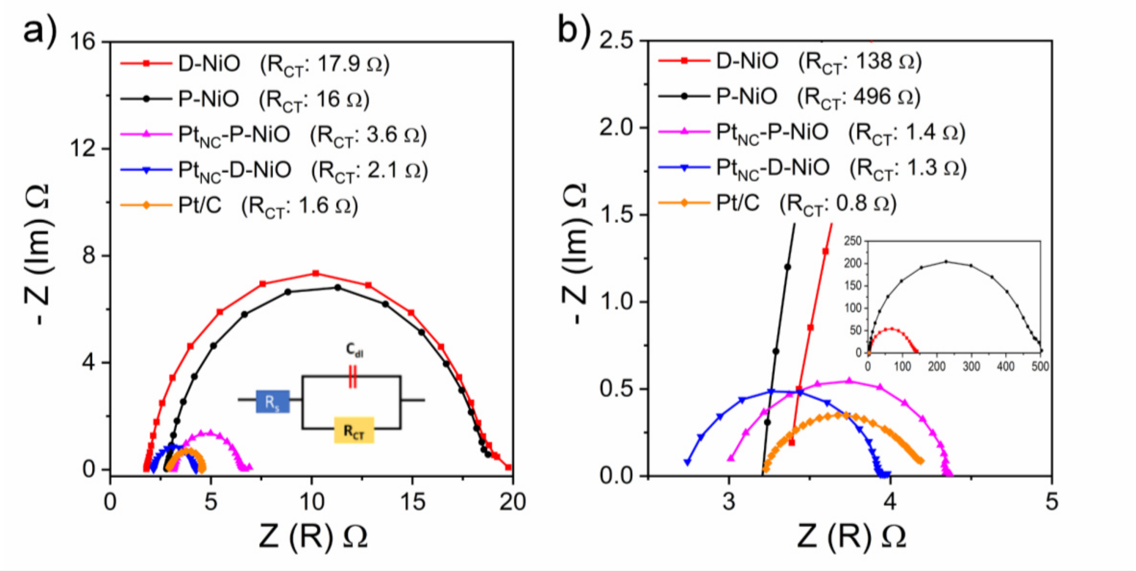

Figure S12. Nyquist plot for HER at (a) 454 mV overpotential in 1 M KOH and (b) 290 mV overpotential in 0.5 M H_2SO_4.

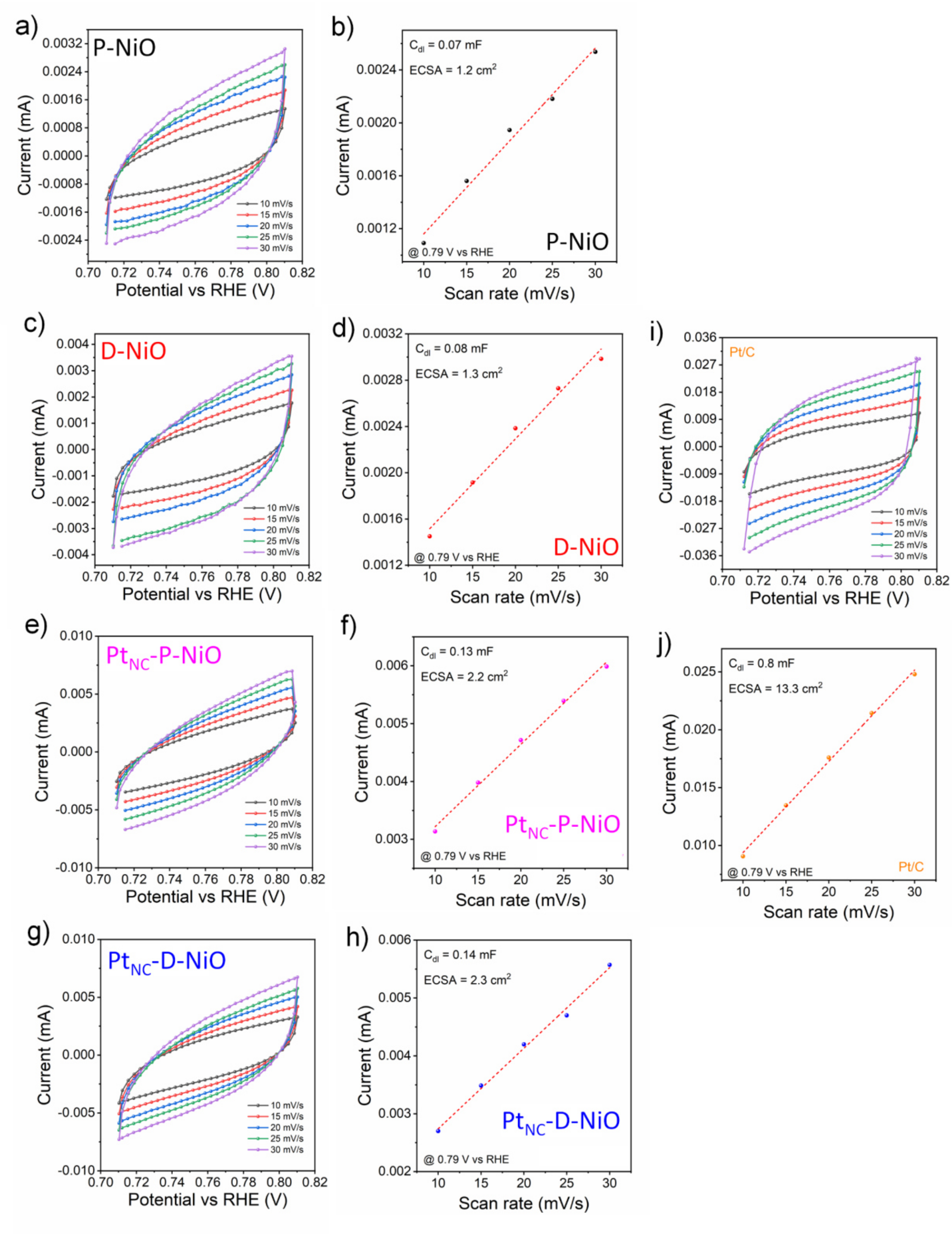

Figure S13. CV plots of (a) P-NiO, (c) D-NiO, (e) Pt_{NC}-P-NiO, (g) Pt_{NC}-D-NiO and (i) Pt/C at different scan rates. Current (recorded at a fixed potential) as a function of scan rate for (b) P-NiO, (d) D-NiO, (f) Pt_{NC}-P-NiO, (h) Pt_{NC}-D-NiO and (j) Pt/C.

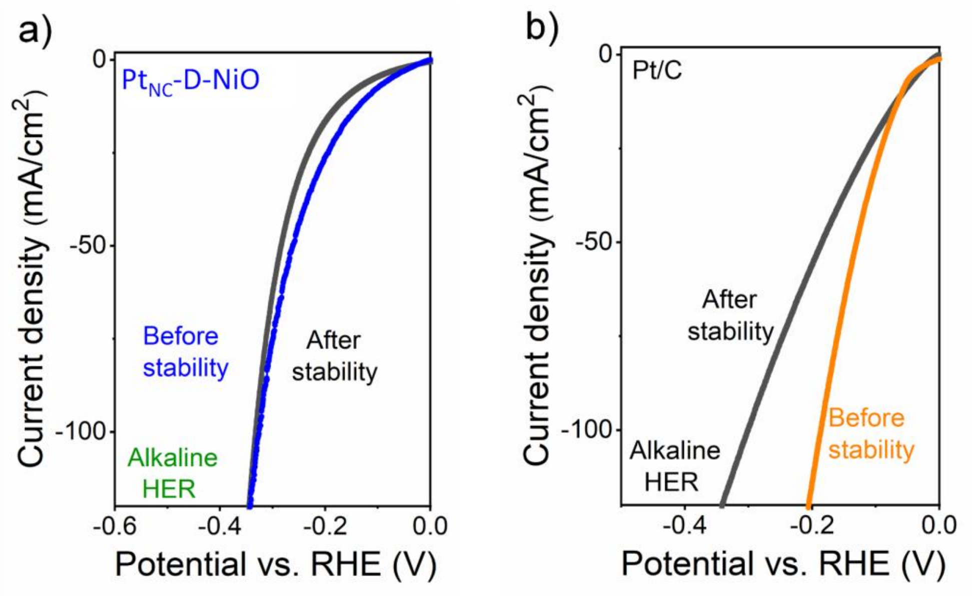

Figure S14. Alkaline HER LSV polarization curves before and after the stability test of (a) Pt_{NC}-D-NiO and (b) Pt/C.

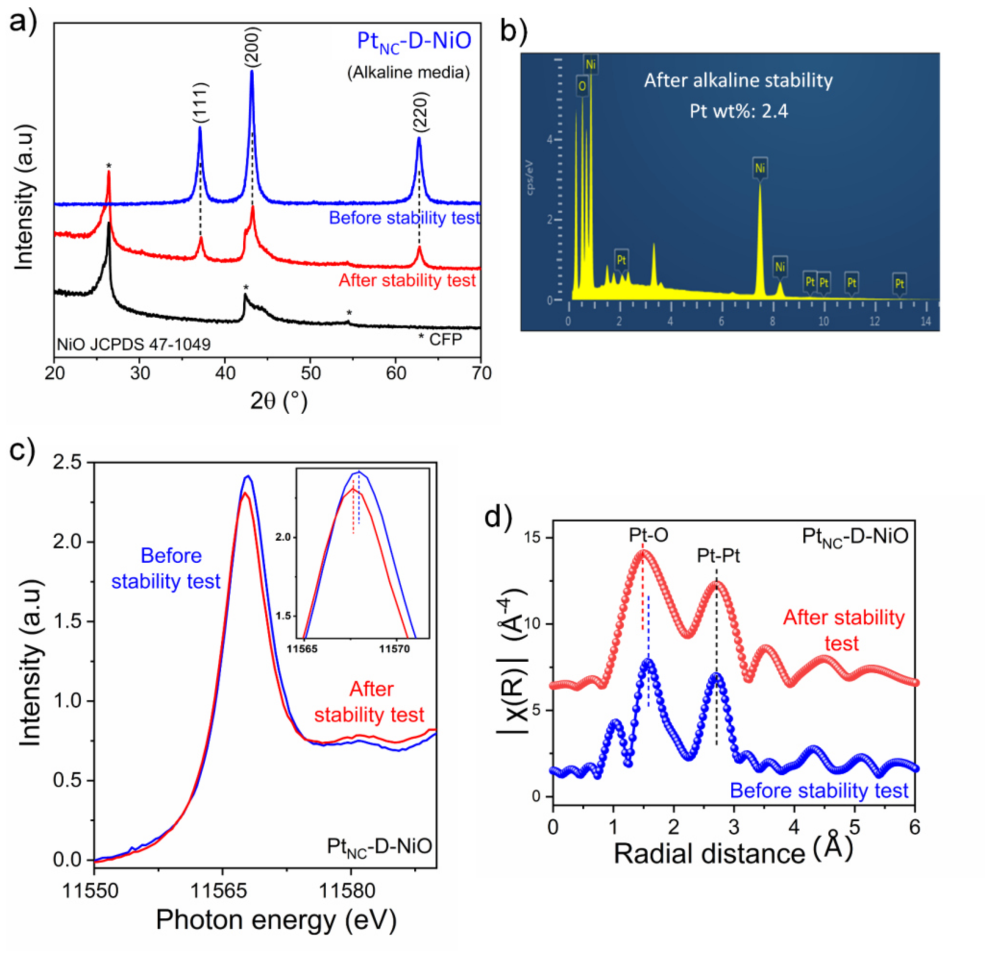

Figure S15. (a) XRD pattern and (b) EDS pattern of Pt$_{NC}$-D-NiO after the stability test in alkaline medium. Experimental Pt-L$_3$ edge (c) XANES and (d) FT-EXAFS spectra of Pt$_{NC}$-D-NiO after the stability test in alkaline medium.

The slight shortening of the Pt-O bond length observed after the stability test suggests the progressive removal of surface oxygen species under reductive conditions, while the persistence of Pt-O$_{support}$ coordination indicates robust interfacial integration between the Pt nanoclusters and the NiO support (Figure S15d).

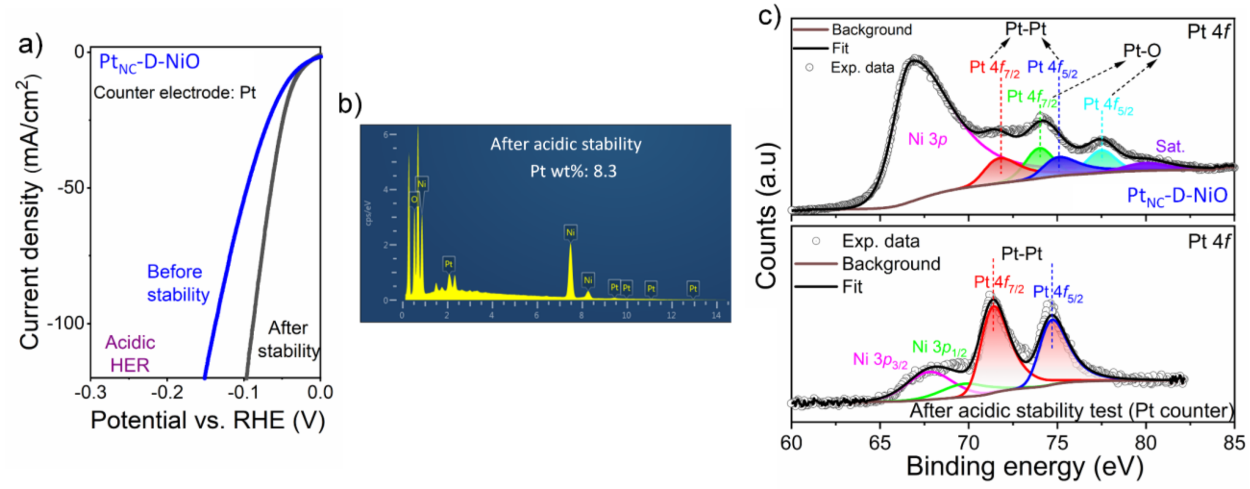

Figure S16. (a) Acidic HER LSV polarization curves before and after the stability test of Pt_{NC}-D-NiO with Pt as the counter electrode. (b) EDS pattern of Pt_{NC}-D-NiO after the stability test in acidic medium. (c) High-resolution Pt 4f XPS spectra of Pt_{NC}-D-NiO before and after the stability test in acidic medium with Pt as the counter electrode.

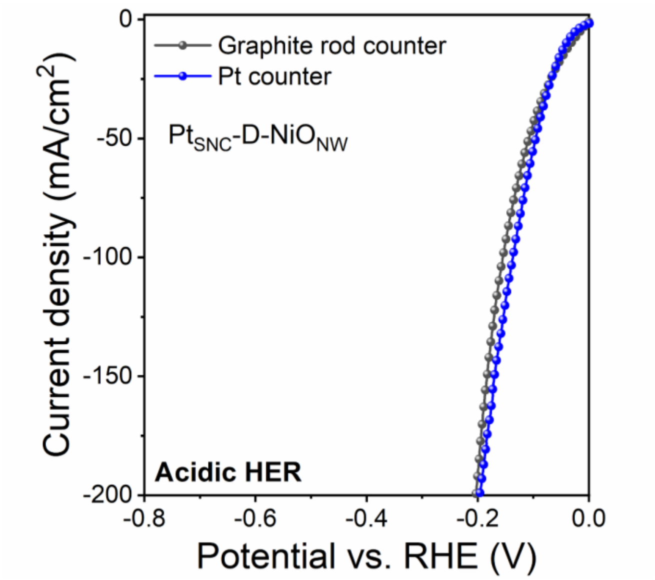

Figure S17. Comparison of the acidic HER LSV polarization curves of Pt_{NC}-D-NiO with Pt and graphite rod as counter electrode.

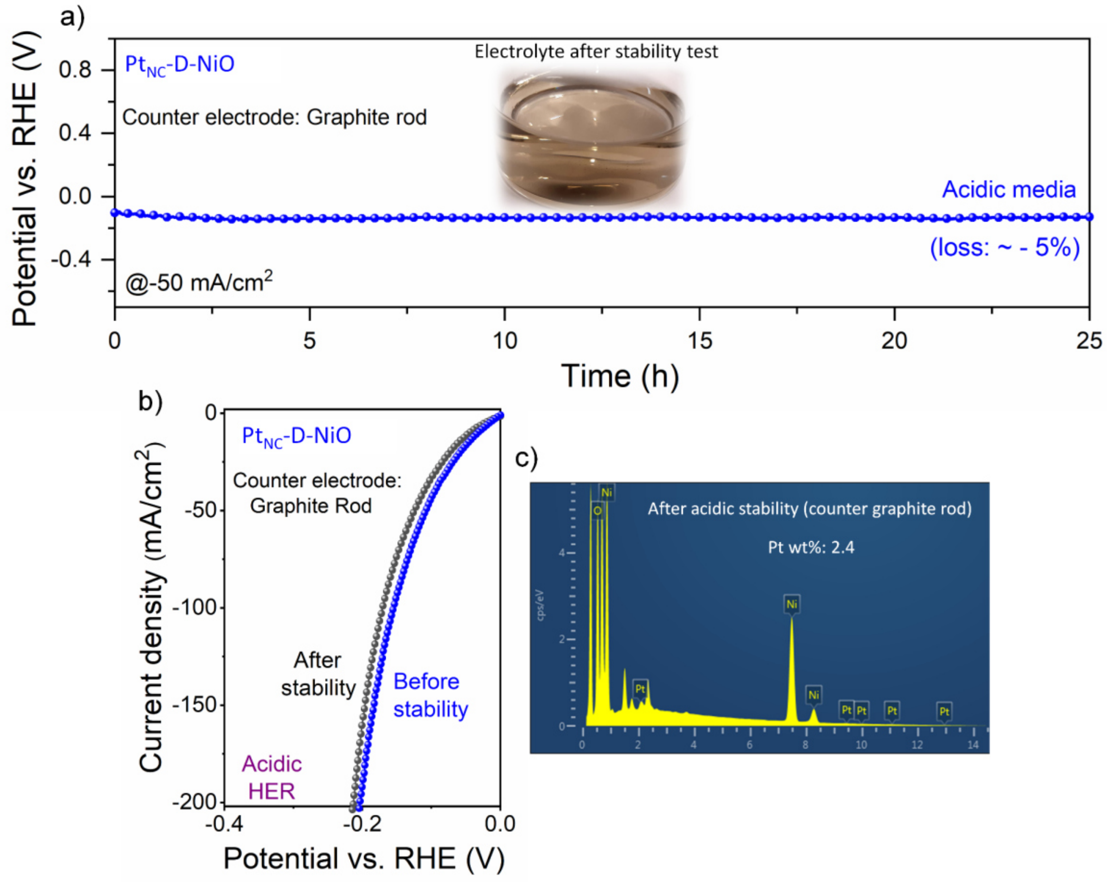

Figure S18. (a) Chronopotentiometric stability test for Pt$_{NC}$-D-NiO with graphite rod as counter electrode in 0.5 M H_2SO_4 at a current density of -50 mA cm^{-2}. The inset shows the electrolyte containing the leached graphite after the acidic stability test. (b) Acidic HER LSV polarization curves before and after the stability test of Pt$_{NC}$-D-NiO with graphite rod as the counter electrode. (c) EDS pattern of Pt$_{NC}$-D-NiO after the stability test in acidic medium with graphite as counter electrode.

Table S3. Activity and stability comparison of with reported literatures.

Catalyst	η@10 mA/cm² (mV)		Stability	Reference
	Acidic	Alkaline		
Pt_{NC}-D-NiO	41	124	150 h	This work
Pt@PCM	105	139	5 h	*Sci. Adv.* **2018**, *4*, eaao6657
$Ru_{SA}CoFe_2$/G		164	24 h	*Energy Environ. Sci.*, **2020**, *13*, 5152
CoN_4-SAC		111		*Adv. Funct. Mater.* **2021**, *31*, 2100547
Mo SAs/ML-MoS_2	107	209		*ACS Nano* **2020**, *14*, 767
Pt-SA decorated VS_2	77		12 h	*ACS Nano* **2020**, *14*, *5600*
Pt_3Ni_2 NWs-S/C		42	5 h	*Nat. Commun.* **2017**, *8*, *14580*
Ru black	150	125		*ACS Catal.* **2018**, *8*, *11094*
Li-$IrSe_2$@CNT	55	72	10 h	*Angew. Chem. Int. Ed.* **2019**, *58*, 14764
Pt-Ni		60	1 h	*J. Mater. Chem. A*, **2016**, *4*, 12392
Mo-SAC	154	132	10 h	*Angew. Chem. Int. Ed.* **2017**, *56*, 16086
Ni_{SA}-MoS_2	110	98		*Nano Energy*, **2018**, *53*, *458*
3D-NiCoP	80	105	15 h	*Nano Res.*, **2019**, *12*, 375
NiFe-NCs		197	18 h	*ACS Appl. Mater. Interfaces*, **2017**, *9*, *41906*
Pt-MoS_2	67		24 h	*Nano Energy*, **2022**, *94*, 106913
Pt@MC-VA	58		24 h	*Mater. Today Energy* **2024**, *43*, 101600

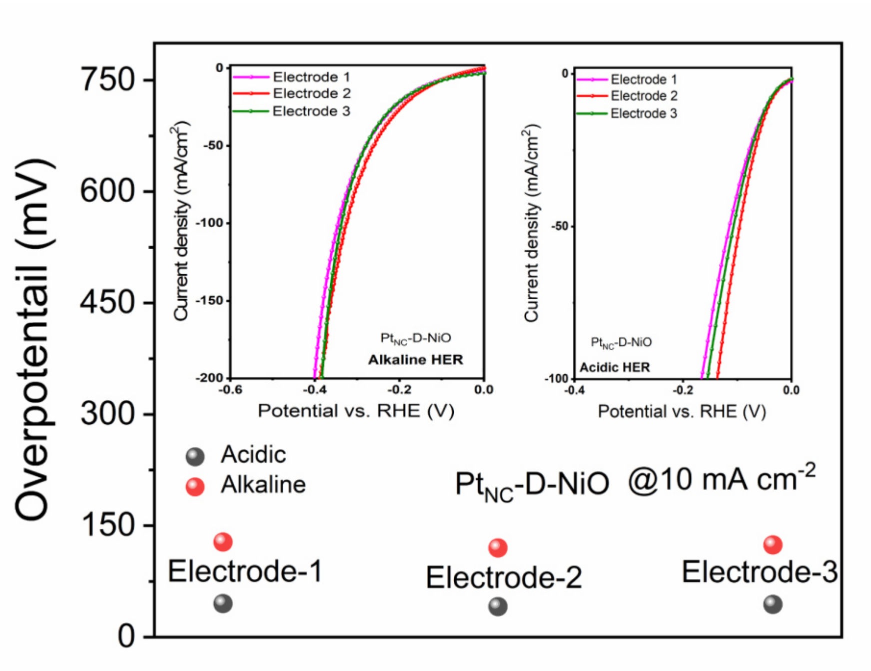

Figure S19. HER LSV polarization curves of three different electrodes for Pt_{NC}-D-NiO in alkaline and acidic media.

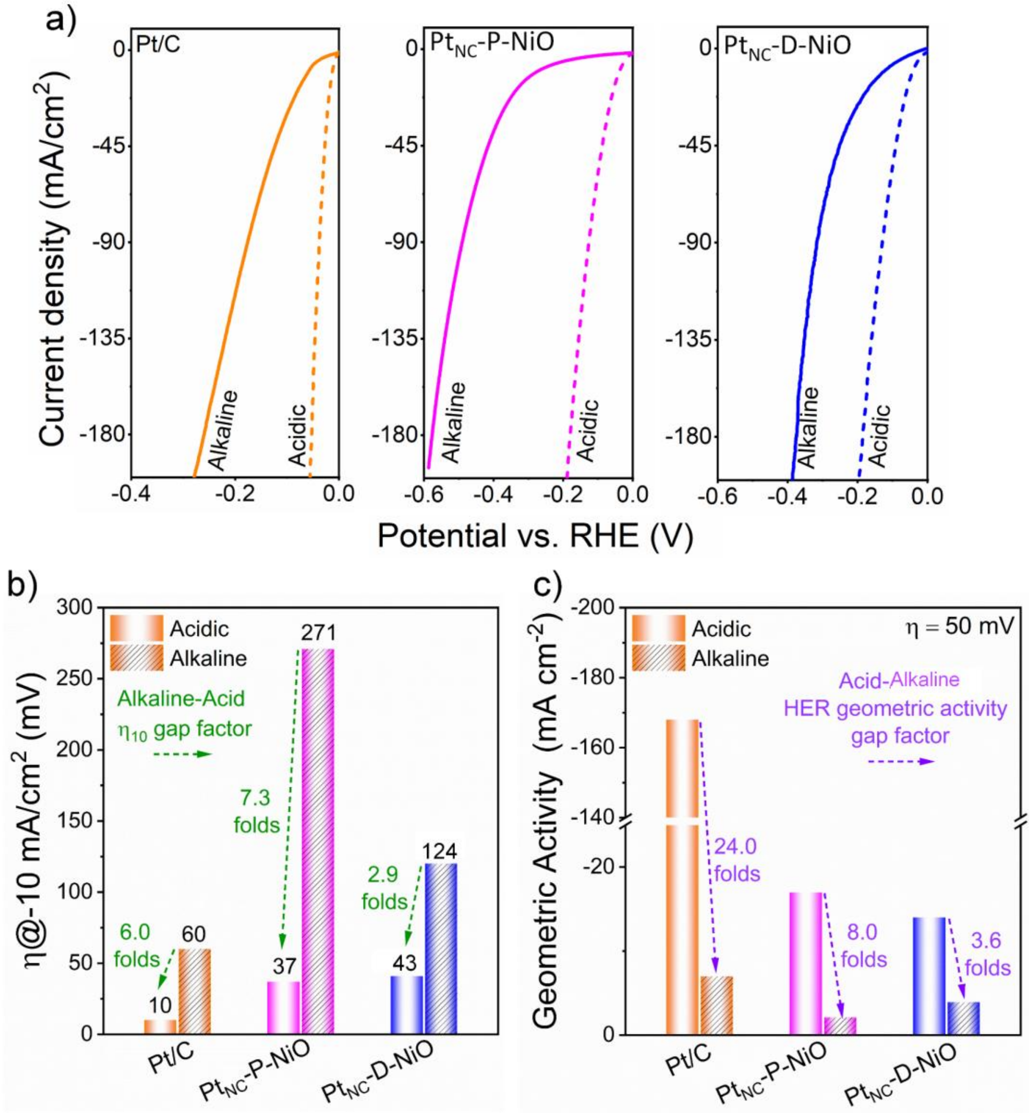

Figure S20. (a) Alkaline and acidic HER LSV polarization curves for Pt/C, Pt_{NC}-P-NiO and Pt_{NC}-D-NiO. (b) Overpotential required to reach a current density of -10 mA cm^{-2} in alkaline and acidic medium for Pt/C, Pt_{NC}-P-NiO and Pt_{NC}-D-NiO. The alkaline-acid η_{10} gap factor can be determined by $\eta_{alkaline}/\eta_{acid}$. (c) Geometric current density reached at 50 mV overpotential in alkaline and acidic medium for Pt/C, Pt_{NC}-P-NiO and Pt_{NC}-D-NiO. The acid-alkaline geometric activity gap factor can be determined by Geometric activity$_{acid}$/Geometric activity$_{alkaline}$.

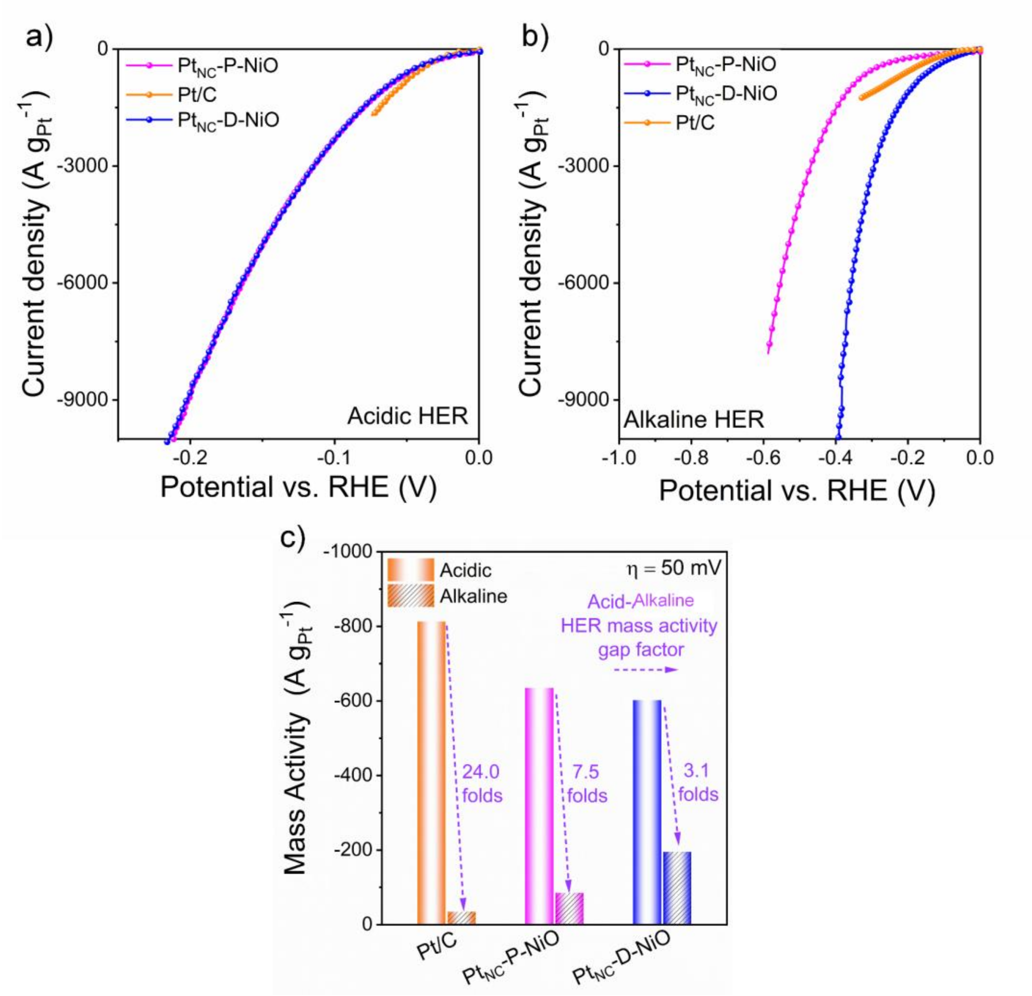

Figure S21. The noble-metal-mass-normalized (a) acidic and (b) alkaline HER LSV polarization curves of Pt/C, Pt_{NC}-P-NiO and Pt_{NC}-D-NiO. (c) Mass activity obtained at 50 mV overpotential in alkaline and acidic medium for Pt/C, Pt_{NC}-P-NiO and Pt_{NC}-D-NiO. The acid-alkaline mass activity gap factor can be determined by Mass $activity_{acid}$/Mass $activity_{alkaline}$.

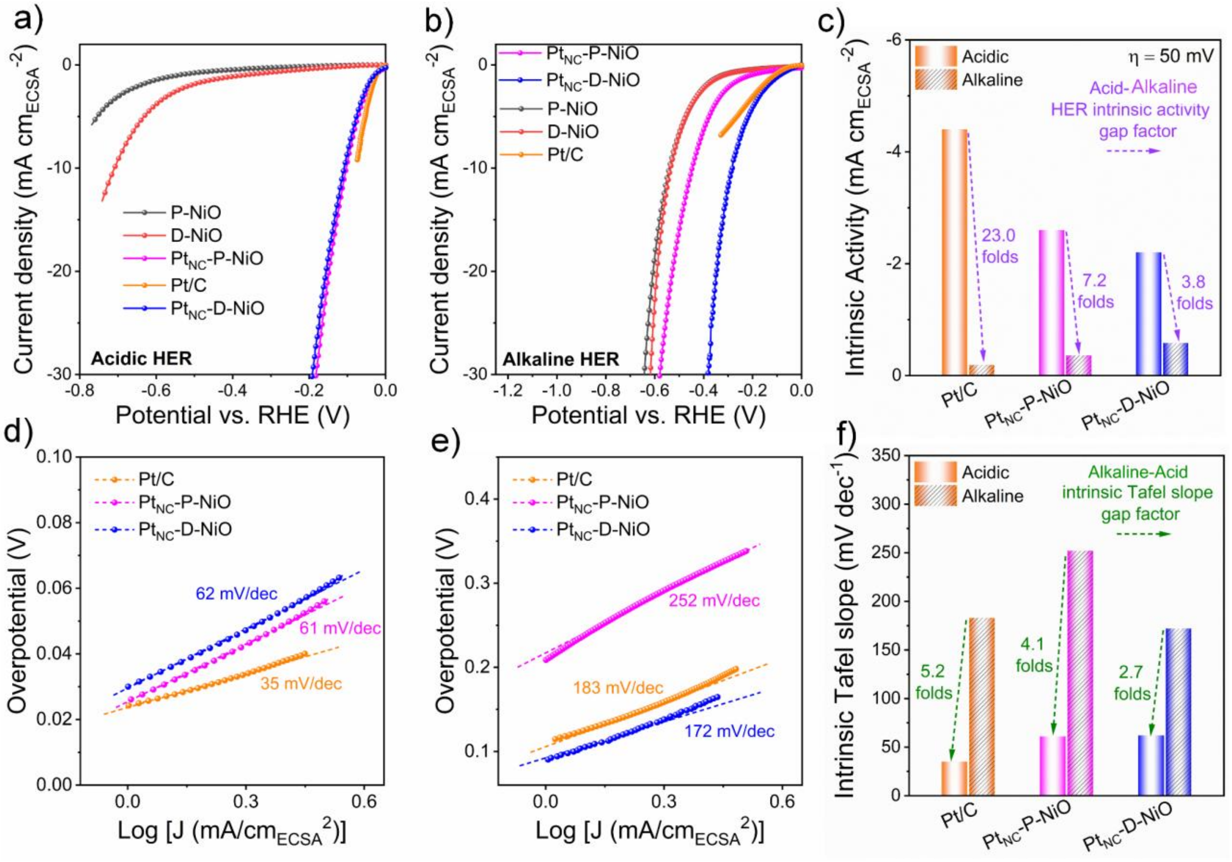

Figure S22. ECSA normalized (a) acidic and (b) alkaline HER LSV polarization curves. (c) Intrinsic activity obtained at 50 mV overpotential in alkaline and acidic medium for Pt/C, Pt_{NC}-P-NiO and Pt_{NC}-D-NiO. The acid-alkaline intrinsic activity gap factor can be determined by Intrinsic $activity_{acid}$/Intrinsic $activity_{alkaline}$. Corresponding intrisic Tafel plots with the kinetics current density normalized by ECSA for Pt/C, Pt_{NC}-P-NiO and Pt_{NC}-D-NiO in (d) acidic and (e) alkaline media. (f) Corresponding intrinsic Tafel slopes in alkaline and acidic medium for Pt/C, Pt_{NC}-P-NiO and Pt_{NC}-D-NiO. The alkaline-acid Intrinsic Tafel slope gap factor can be determined by Intrinsic Tafel $slope_{alkaline}$/Intrinsic Tafel $slope_{acid}$.

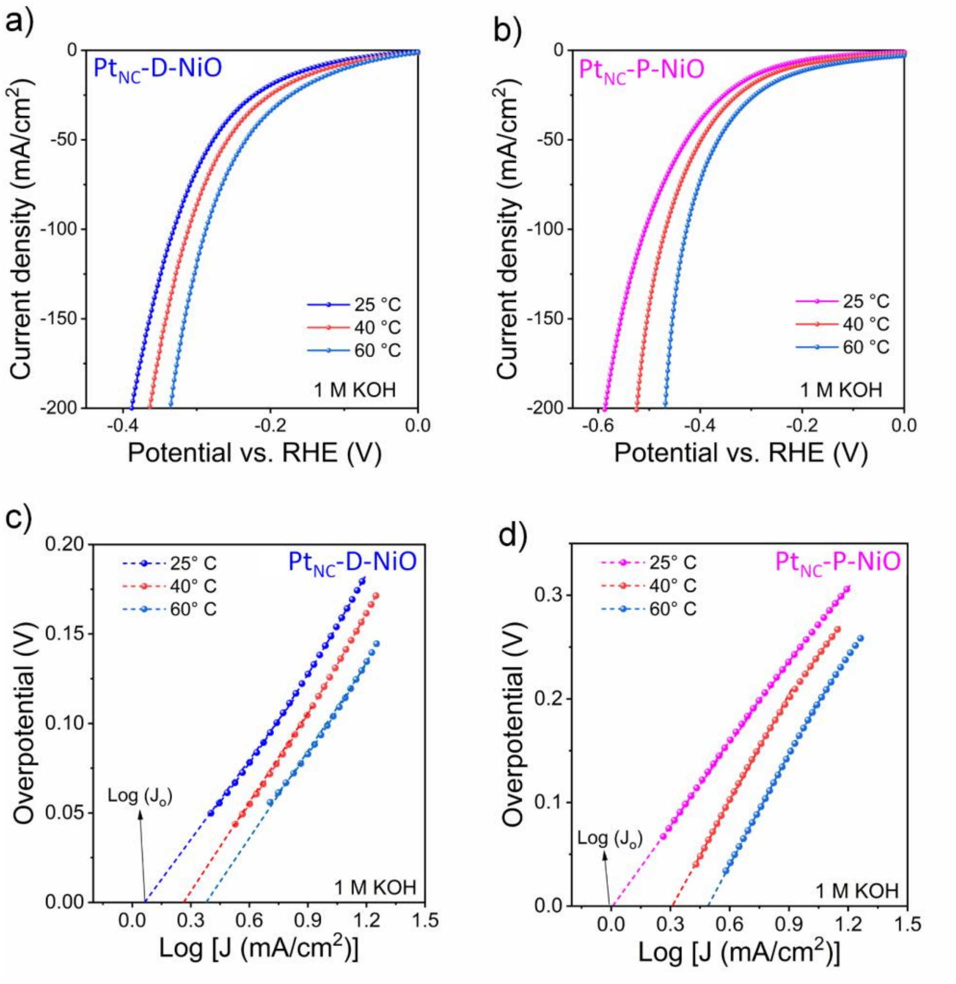

Figure S23. HER LSV curves of (a) Pt$_{NC}$-D-NiO and (b) Pt$_{NC}$-P-NiO in 1 M KOH at 25, 40, and 60 °C. Corresponding Tafel plots of (c) Pt$_{NC}$-D-NiO and (d) Pt$_{NC}$-P-NiO in 1 M KOH at 25, 40, and 60 °C.

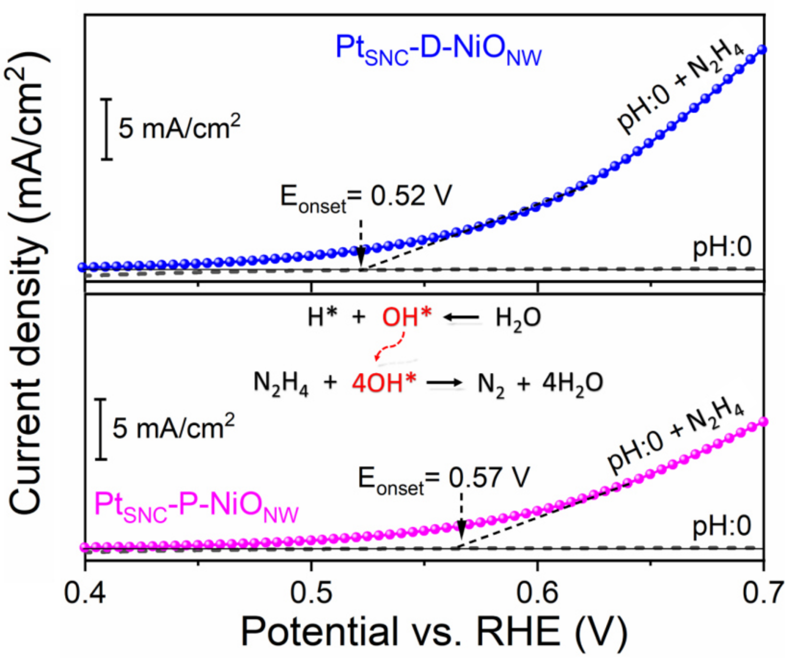

Figure S24. LSV curves of Pt_{NC}-D-NiO and Pt_{NC}-P-NiO in acidic electrolyte containing 0.05 M hydrazine (N_2H_4). The anode current in these curves represents the hydrazine oxidation current. The positions marked by arrows indicate the onset potential of hydrazine oxidation.

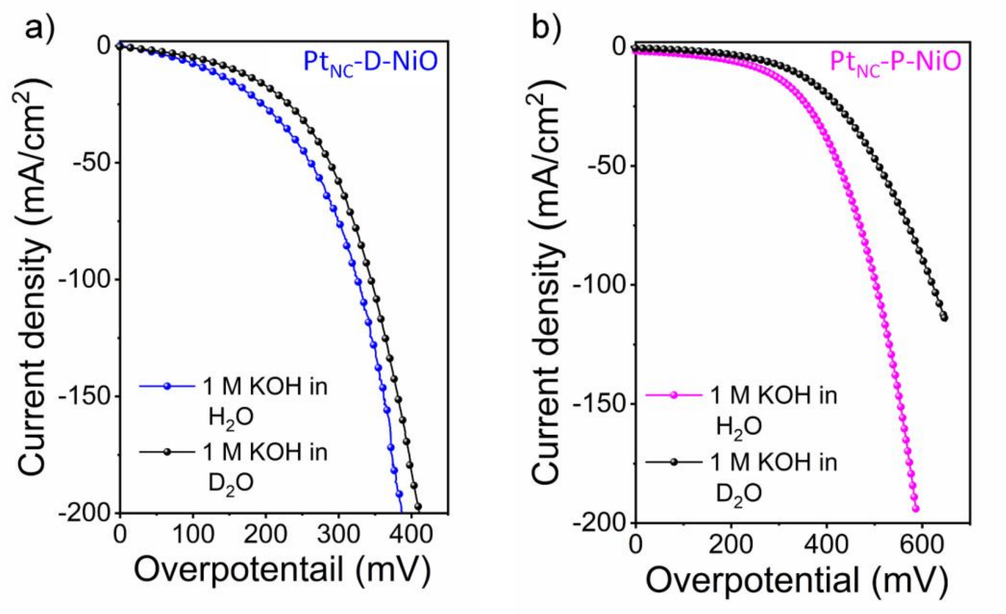

Figure S25. HER LSV curves obtained from (a) Pt_{NC}-D-NiO and (b) Pt_{NC}-P-NiO in 1.0 M KOH-H_2O and 1.0 M KOH-D_2O electrolyte.

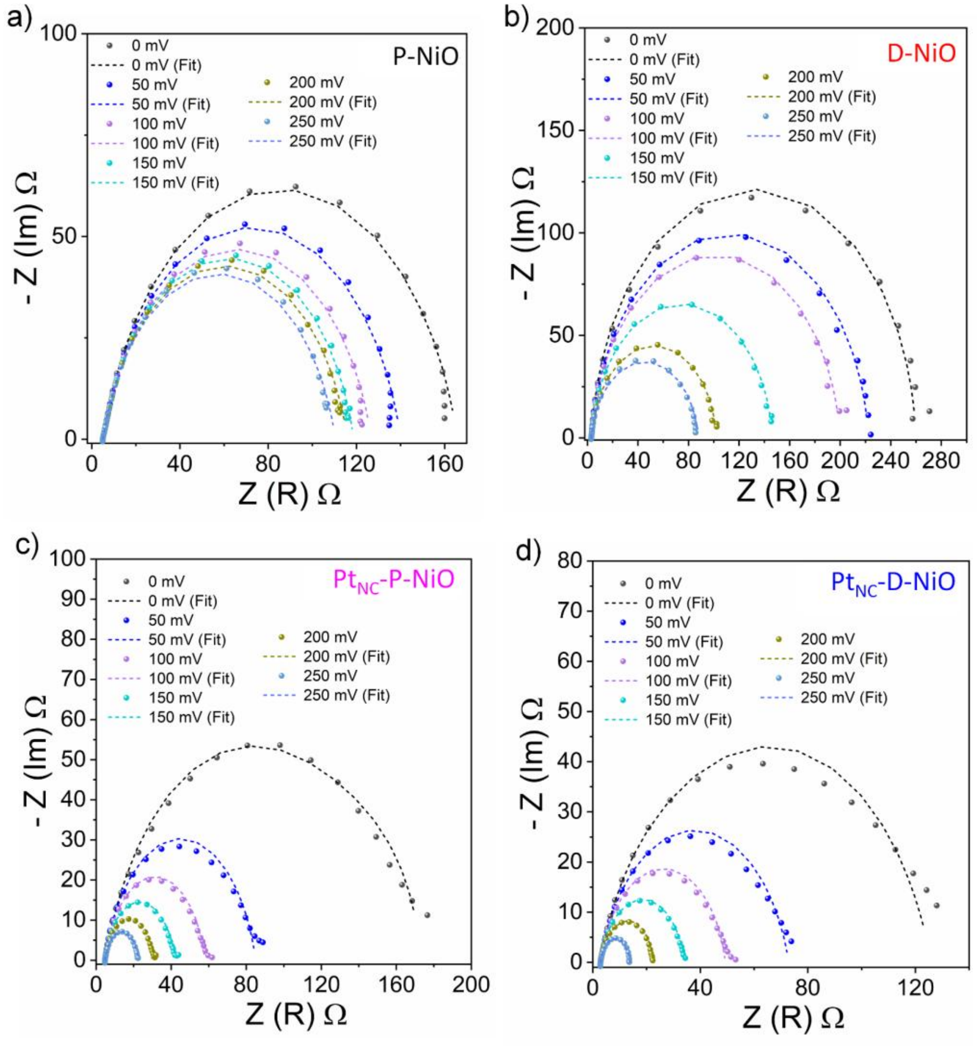

Figure S26. Nyquist plots for (a) P-NiO, (b) D-NiO, (c) Pt$_{NC}$-P-NiO and (d) Pt$_{NC}$-D-NiO in 1 M KOH at various HER overpotentials.

Table S4. The fitted parameters of the EIS data of various catalysts for HER.

Catalyst	η (mV)	R_s (Ω)	Q (F)	R_{ct} (Ω)	C_φ (F)	R_i (Ω)
P-NiO	0	5.082	0.000058	161.2	0.0040	461.8
	50	5.072	0.000051	135.5	0.0047	399.2
	100	5.086	0.000061	121.7	0.015	352.1
	150	5.084	0.000063	114.4	0.031	287.6
	200	5.075	0.000067	110.9	0.064	211.2
	250	4.965	0.000061	103.7	0.097	164.3
D-NiO	0	3.111	0.000066	259.5	0.0029	571.9
	50	3.122	0.000072	218.8	0.0046	423.5
	100	3.12	0.000077	196.8	0.013	362.2
	150	3.094	0.000073	142	0.042	312.75
	200	3.052	0.000076	98.83	0.096	260.9
	250	3.122	0.000075	83.79	0.156	205.3
Pt_{NC}-P-NiO	0	4.561	0.000231	165.8	0.0042	267.3
	50	4.665	0.000141	79.78	0.0070	223.2
	100	4.715	0.000115	53.32	0.084	184
	150	4.708	0.000098	42.95	0.121	125.4
	200	4.751	0.0001	26.15	0.197	94.14
	250	4.779	0.000085	17.54	0.221	73.64
Pt_{NC}-D-NiO	0	2.864	0.000536	123	0.0023	112.064
	50	2.85	0.000336	70.09	0.0465	92.75
	100	2.819	0.000246	47.1	0.152	67.45
	150	2.882	0.000226	31.3	0.211	50.93
	200	2.974	0.000173	19.78	0.268	41.35
	250	2.631	0.000102	11.09	0.315	38.37

Discussion S1:

Calculation of H* coverage on Pt_{NC} ($\Theta_{Pt\text{-}NC}$) for Pt_{NC}-P-NiO:

$Q_{Total\_Pt\text{-}NC\text{-}P\text{-}NiO}$: Total H* adsorption charge for Pt_{NC}-P-NiO (integrating C_φ vs. η profiles for Pt_{NC}-P-NiO in Fig. 6d).

$Q_{Total\_P\text{-}NiO}$: Total H* adsorption charge for P-NiO (integrating C_φ vs. η profiles for P-NiO in Fig. 6d).

$Q_{Total\_Pt\text{-}NC} = Q_{Total\_Pt\text{-}NC\text{-}P\text{-}NiO} - Q_{Total\_P\text{-}NiO}$: Total H* adsorption charge on Pt_{NC} for Pt_{NC}-P-NiO.

$S_{Total\_Pt\text{-}NC}$: Total surface area of Pt_{NC} in Pt_{NC}-P-NiO.

$\mathbf{Q_{Unit\text{-}Pt\text{-}NC}}$: H* adsorption charge on unit area of Pt_{NC} for Pt_{NC}-P-NiO (assuming all Pt_{NC} contribute to the H* adsorption in HER).

$$\mathbf{Q_{Unit\text{-}Pt\text{-}NC}} = Q_{Total\_Pt\text{-}NC} / S_{Total\_Pt\text{-}NC}$$

<u>Total surface area of Pt_{NC}:</u>

$S_{Total\_Pt\text{-}NC}$ = Total number of Pt_{NC} x Surface area of single Pt_{NC}

= (Total volume of Pt_{NC} / Volume of single Pt_{NC}) x Surface area of single Pt_{NC}

$$= \frac{3m_{Pt}}{\rho_{Pt}\, R_{Pt}}$$

m_{Pt} : Mass of Pt_{NC} in Pt_{NC}-P-NiO = Mass of loaded catalysts x Pt_{NC} wt%

= 0.36 mg x 2.41 wt% = 8.7 x 10^{-6} g

ρ_{Pt} : Density of Pt_{NC} (21.5 g/cm^3);

R_{Pt} : Radius of Pt_{NC} in Pt_{NC}-P-NiO (1.25 nm)

$$\mathbf{S_{Total\_Pt\text{-}NC}} = \frac{3m_{Pt}}{\rho_{Pt}\, R_{Pt}} = 9.7\ cm^2$$

$\mathbf{Q_{Total\_Pt\text{-}NC}} = Q_{Total\_Pt\text{-}NC\text{-}P\text{-}NiO} - Q_{Total\_P\text{-}NiO} = 26080 - 8260\ \mu C = 17820\ \mu C$

$\mathbf{Q_{Unit\text{-}Pt\text{-}NC}} = Q_{Total\_Pt\text{-}NC} / S_{Total\_Pt\text{-}NC} = 17820 / 9.7 = 1837\ \mu C/cm^2$

Assuming the H* adsorption charge for a monolayer for a Pt (111) surface, i.e. $Q_{standard}$ = 210 μC/cm^2, the apparent H* coverage of Pt_{NC} for Pt_{NC}-P-NiO ($\Theta_{Pt\text{-}NC:\ Pt\text{-}NC\text{-}P\text{-}NiO}$) in equivalent monolayers of H* could be calculated as follow:

$\mathbf{\Theta_{Pt\text{-}NC:\ Pt\text{-}NC\text{-}P\text{-}NiO}} = Q_{Unit\text{-}Pt\text{-}NC} / 210 = 1837 / 210 = 8.7$

<u>Discussion S2:</u>

Calculation of H* coverage on Pt_{NC} ($\Theta_{Pt\text{-}NC}$) for Pt_{NC}-D-NiO:

$Q_{Total\_Pt\text{-}NC\text{-}D\text{-}NiO}$: Total H* adsorption charge for Pt_{NC}-D-NiO (integrating C_{φ} vs. η profiles for Pt_{NC}-D-NiO in Fig. 6d).

$Q_{Total\_D\text{-}NiO}$: Total H* adsorption charge for D-NiO (integrating C_{φ} vs. η profiles for D-NiO in Fig. 6d).

$Q_{Total\_Pt\text{-}NC} = Q_{Total\_Pt\text{-}NC\text{-}D\text{-}NiO} - Q_{Total\_D\text{-}NiO}$: Total H* adsorption charge on Pt_{NC} for Pt_{NC}-D-NiO.

$S_{Total\_Pt\text{-}NC}$: Total surface area of Pt_{NC} in Pt_{NC}-D-NiO.

$\mathbf{Q_{Unit\text{-}Pt\text{-}NC}}$: H* adsorption charge on unit area of Pt_{NC} for Pt_{NC}-D-NiO (assuming all Pt_{NC} contribute to the H* adsorption in HER).

$$\mathbf{Q_{Unit\text{-}Pt\text{-}NC}} = Q_{Total\_Pt\text{-}NC} / S_{Total\_Pt\text{-}NC}$$

<u>Total surface area of Pt_{NC}:</u>

$S_{Total\_Pt\text{-}NC}$ = Total number of Pt_{NC} x Surface area of single Pt_{NC}

= (Total volume of Pt_{NC} / Volume of single Pt_{NC}) x Surface area of single Pt_{NC}

$$= \frac{3m_{Pt}}{\rho_{Pt}\, R_{Pt}}$$

m_{Pt} : Mass of Pt_{NC} in Pt_{NC}-D-NiO = Mass of loaded catalysts x Pt_{SNC} wt%

= 0.36 mg x 2.35 wt% = 8.5 x 10^{-6} g

ρ_{Pt} : Density of Pt_{SNC} (21.5 g/cm^3);

R_{Pt} : Radius of Pt_{NC} in Pt_{NC}-D-NiO (1.1 nm)

$$\mathbf{S_{Total\_Pt\text{-}NC}} = \frac{3m_{Pt}}{\rho_{Pt}\, R_{Pt}} = 10.8\ cm^2$$

$\mathbf{Q_{Total\_Pt\text{-}NC}} = Q_{Total\_Pt\text{-}NC\text{-}D\text{-}NiO} - Q_{Total\_D\text{-}NiO} = 41807 – 11752\ \mu C = 30055\ \mu C$

$\mathbf{Q_{Unit\text{-}Pt\text{-}NC}} = Q_{Total\_Pt\text{-}NC} / S_{Total\_Pt\text{-}NC} = 30055 / 10.8 = 2782.9\ \mu C/cm^2$

Assuming the H* adsorption charge for a monolayer for a Pt (111) surface, i.e. $Q_{standard}$ = 210 μC/cm^2, the apparent H* coverage of Pt_{NC} for Pt_{NC}-D-NiO ($\Theta_{Pt\text{-}SNC}$: $_{Pt\text{-}NC\text{-}D\text{-}NiO}$) in equivalent monolayers of H* could be calculated as follow:

$\mathbf{\Theta_{Pt\text{-}NC}: {}_{Pt\text{-}NC\text{-}D\text{-}NiO}} = Q_{Unit\text{-}Pt\text{-}NC} / 210 = 2782.9 / 210 = 13.2$
